# Isolating pulmonary microvascular endothelial cells *ex vivo*: Implications for pulmonary arterial hypertension, and a caution on the use of commercial biomaterials

**DOI:** 10.1371/journal.pone.0211909

**Published:** 2019-02-27

**Authors:** Bradley M. Wertheim, Yi-Dong Lin, Ying-Yi Zhang, Andriy O. Samokhin, George A. Alba, Elena Arons, Paul B. Yu, Bradley A. Maron

**Affiliations:** 1 Department of Medicine, Division of Pulmonary and Critical Care Medicine, Brigham and Women’s Hospital, Boston, MA, United States of America; 2 Department of Medicine, Division of Cardiovascular Medicine, Brigham and Women’s Hospital, Boston, MA, United States of America; 3 Department of Medicine, Division of Pulmonary and Critical Care Medicine, Massachusetts General Hospital, Boston, MA, United States of America; Vanderbilt University Medical Center, UNITED STATES

## Abstract

Transcriptomic analysis of pulmonary microvascular endothelial cells from experimental models offers insight into pulmonary arterial hypertension (PAH) pathobiology. However, culturing may alter the molecular profile of endothelial cells prior to analysis, limiting the translational relevance of results. Here we present a novel and validated method for isolating RNA from pulmonary microvascular endothelial cells (PMVECs) *ex vivo* that does not require cell culturing. Initially, presumed rat PMVECs were isolated from rat peripheral lung tissue using tissue dissociation and enzymatic digestion, and cells were cultured until confluence to assess endothelial marker expression. Anti-CD31, anti-von Willebrand Factor, and anti-α-smooth muscle actin immunocytochemistry/immunofluorescence signal was detected in presumed rat PMVECs, but also in non-endothelial cell type controls. By contrast, flow cytometry using an anti-CD31 antibody and isolectin 1-B_4_ (from *Griffonia simplicifolia*) was highly specific for rat PMVECs. We next developed a strategy in which the addition of an immunomagnetic selection step for CD31+ cells permitted culture-free isolation of rat PMVECs *ex vivo* for RNA isolation and transcriptomic analysis using fluorescence-activated cell sorting. Heterogeneity in the validity and reproducibility of results using commercial antibodies against endothelial surface markers corresponded to a substantial burden on laboratory time, labor, and scientific budget. We demonstrate a novel protocol for the culture-free isolation and transcriptomic analysis of rat PMVECs with translational relevance to PAH. In doing so, we highlight wide variability in the quality of commonly used biological reagents, which emphasizes the importance of investigator-initiated validation of commercial biomaterials.

## Introduction

Pulmonary arterial hypertension (PAH) is a severe cardiopulmonary disease characterized by dysregulated transcriptional mechanisms that promote endothelial dysfunction [1]. Studying pulmonary artery endothelial cells (PAECs) from PAH patients is optimal, but access is limited, in part, by low disease prevalence and technical obstacles [2,3]. Therefore, studying PAECs from PAH animal models offers an important and well-established alternative approach to analyzing disease-specific pathobiological mechanisms [4]. Protocols for isolating primary PAECs from PAH models have been reported previously, but these strategies require passaging cells *in vitro* to ensure a sufficient population for further analysis [5–19]. However, sequential passaging may alter the phenotype and molecular program of cells [20]. Effective cell isolation without serial passaging is possible,[16] but has not been reported for rodent PAECs.

Limited reproducibility of published scientific results has led to an emerging initiative among funding sponsors, including the National Institutes of Health, that emphasizes data quality [21,22]. The widespread availability of commercial biomedical products has simplified reagent preparation and improved laboratory efficiency. However, inconsistent product quality—for example, uncertain binding epitopes among some commercial antibodies—may contribute to variability in experimental biology. In turn, data validating these biotechnologies is likely to improve rigor of scientific findings, but is rarely reported independently by investigators [4,23–26].

Microvascular endothelial dysfunction has been implicated in multiple aspects of PAH pathobiology including angiogenesis, proliferation, apoptosis, and adaptation to shear stress [5,27,28]. In this report, we describe a practical method for isolating high-quality mRNA for transcriptomic analysis from rat pulmonary microvascular endothelial cells (PMVECs) *ex vivo* without cell passaging. In doing so, we also demonstrate wide variability in the quality of purchased laboratory reagents. Together, the current work outlines a cell culture-free approach to studying PMVECs, and reinforces the use of commercial biomaterials without on-site validation as a modifiable step toward enhancing the reproducibility of data in PAH.

## Methods and results

### Cell culture and reagents

Human pulmonary artery endothelial cells (HPAECs), human lung fibroblasts (HLFs), and human pulmonary artery smooth muscle cells (HPASMCs) were purchased from Lonza. Rat pulmonary artery endothelial cells (RPAECs), rat pulmonary artery smooth muscle cells (RPASMCs), and rat lung fibroblasts (RLFs) were purchased from Cell Biologics. The details of each cell type are provided in the **S1 Supporting Information.** Rat PMVECs were harvested in our laboratory from the peripheral region of the lung, expressed CD31 (Santa Cruz 376764 [Ab #1] for immunofluorescence and immunohistochemistry; R & D Biosystems FAB3628P [Ab #20] for flow cytometry), and, in some experiments were confirmed by co-labeling with isolectin 1-B_4_ from *Griffonia simplicifolia* (GS-IB_4_) (Thermo Fisher, Catalog #I21411). Cell culture was performed under standard conditions (37°C, 5% CO_2_, 90% humidity) in vendor-recommended media (**S1 Supporting Information**). Cell passages 1–8 were used for experiments. Details on commercially purchased antibodies (Abs #1–22) used for experiments are presented in **Table 1.**

**Table 1:** Characteristics of antibodies used in experiments.

**Number**	**Target**	Vendor	Catalog #	Isotype	Reactivity	Clonality (Clone #)	Epitope/Immunogen (if available)	Conjugate	Vendor Predicted use
1	CD31	Santa Cruz	376764	M, IgG_1_	M, R, H	Monoclonal (H-3)	Epitope: aa 699–727 of mouse CD31	-	WB, IP, IF, IHC/ICC, ELISA
2	CD31	Becton Dickinson Biosciences	550300	M, IgG_1_κ	R, P	Monoclonal (TLD-3A12)	Immunogen: Lewis rat microglia Epitope not mapped	-	FC, IHC, IP, ELISA
3	Human IgG	Abcam	109489	Rab, IgG	H	Monoclonal (EPR4421)	Immunogen: proprietary synthetic peptide within aa 150–250. Epitope: within aa 155–185 of human IgG	-	WB, IHC
4	Mouse IgG	Vector Laboratories	BA-2000	H, IgG	M	Polyclonal	Epitope not mapped	Biotin	IHC, IF, ELISA
5	Rabbit IgG	Vector Laboratories	BA-1000	G, IgG	Rab	Polyclonal	Epitope not mapped	Biotin	IHC, IF, ELISA
6	vWF	Abcam	6994	Rab, IgG	R, S, GP, Co, D, H, P	Polyclonal	Immunogen: full length native human vWF Epitope not mapped	-	IF/ICC, IHC, WB, FC
7	α-SMA	Abcam	5694	Rab, IgG	M, R, C, GP, Co, D, H, P	Polyclonal	Immunogen: synthetic peptide corresponding to N-terminus of human α-SMA, aa 1–100 Epitope not mapped	-	IF/ICC, IHC, WB, ELISA
8	RECA-1	Santa Cruz	52665	M, IgG_1_κ	M, R	Monoclonal (RECA-1)	Immunogen: peripheral and mesenteric lymph nodes of AO rat origin Epitope proprietary	-	WB, IP, IF
9	Vimentin	Abcam	92547	Rab, IgG	M, R, H, RM	Monoclonal (EPR3776)	Immunogen: proprietary synthetic peptide within aa 400 to the C-terminus Epitope: within aa 440–470 of human vimentin	-	WB, FC, IHC, IF
10	Rabbit IgG	Abcam	150079	G, IgG	Rab	Polyclonal	Epitope not mapped	AF 647	IHC, IF, ELISA, FC
11	Mouse IgG	Abcam	150113	G, IgG	M	Polyclonal	Epitope not mapped	AF 488	IHC, IF, ELISA, FC
12	CD31	Becton Dickinson Biosciences	555027	M, BALB/c IgG_1_κ	R, P	Monoclonal (TLD-3A12)	Immonogen: Lewis rat microglia Epitope not mapped	PE	FC
13	CD144	Thermo Fisher	53-1449-42	M, IgG_1_	H	Monoclonal (16B1)	Epitope not mapped	AF 488	FC, IF
14	Isotype control	Santa Cruz	3890 AF 488	M, BALB/cJ IgG_1_	-	Monoclonal (MOPC-31C)	-	AF 488	FC
15	Isotype control	Becton Dickinson Biosciences	550617	M, BALB/c IgG_1_κ	-	Monoclonal (MOPC-31C)	-	PE	FC
16	CD31	Santa Cruz	376764 AF488	M, IgG_1,_ κ	M, R, H	Monoclonal (H-3)	Epitope: aa 699–727 at C-terminus of mouse CD31	AF 488	IF, FC
17	CD144	BioLegend	348507	M, IgG_2a_κ	H	Monoclonal (BV9)	Epitope: EC3-EC4 region in the extracellular domain of human CD144	APC	FC
18	Isotype control	BioLegend	400221	M, IgG_2a_κ	-	Monoclonal (MOPC-173)	-	APC	FC
19	CD144	Santa Cruz	9989 AF488	M, IgG_1_κ	M, R, H, P	Monoclonal (F-8)	Epitope: aa 768–784 of human CD144	AF 488	FC, IF,
20	CD31	R&D Biosystems	FAB3628P	G, IgG	M, R	Polyclonal (lot ABKBO114121)	Immunogen: Glu18-Lys590 of mouse myeloma cell line NS0-derived recombinant CD31 Epitope not mapped	PE	FC
21	Isotype control	R&D Biosystems	IC108P	G, IgG	-	Polyclonal (lot LVD1317031)	-	PE	FC
22	CD31	Thermo Fisher	MA1-80069	M, IgG_1_	NHP, R	Monoclonal (TLD-3A12)	Immunogen: Lewis rat microglia Epitope not mapped	-	ELISA, FC, IF, IHC

aa, amino acid; AF 488, Alexa Fluor 488; AF 647, Alexa Fluor 647; APC, allophycocyanin; C, chicken; Co, cow; D, dog; FC, flow cytometry; G, goat; GP, guinea pig; H, human; Ho, horse; IF, immunofluorescence; IHC, immunohistochemistry; IP, immunoprecipitation; M, mouse; NHP, non-human primate; P, pig; PE, phycoerytherin; R, rat; Rab, rabbit; RM, rhesus monkey; S, sheep; WB, western blot.

### Statistical methods

All statistical analyses were performed using Origin Pro 2015 version b9.2.272. The unpaired Student’s t-test was used for experiments involving comparisons between two samples and one-way analysis of variance (ANOVA) was used for comparisons involving three or more samples. *Post-hoc* analyses were performed using the method of Tukey. Data are presented as mean ± SE. P < 0.05 was used to define statistical significance. Initial data review of immunocytochemistry, immunofluorescence, and flow cytometry experiments was performed by a blinded investigator whenever possible. At least three technical or biological replicates were used per experiment. For colocalization, data were measured in at least 3 cells/field in at least 3 randomly-selected fields/technical replicate.

### Cell isolation from experimental PAH *in vivo*

**Fig 1:**
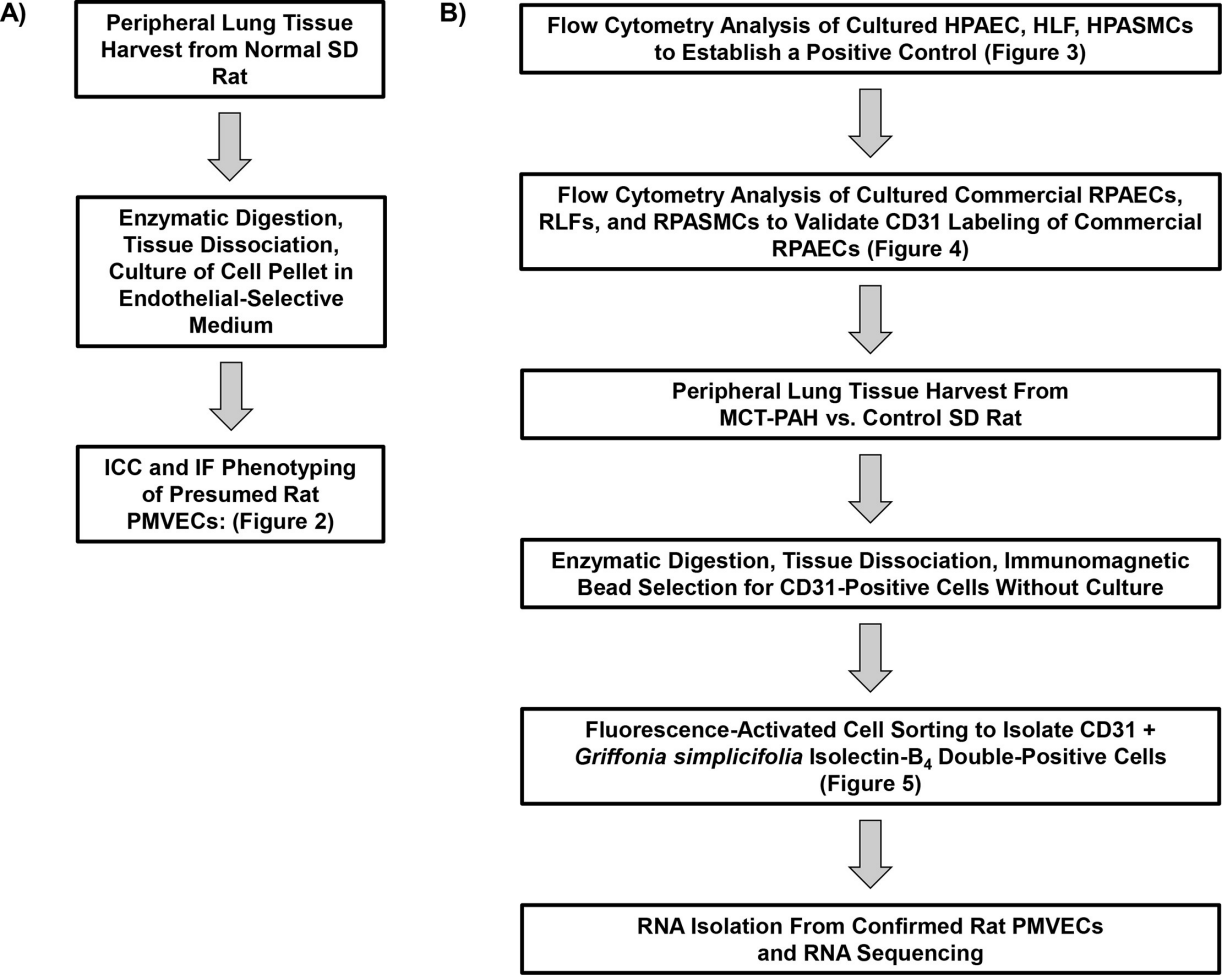
Overall approach to the isolation and phenotyping of rat pulmonary microvascular endothelial cells (PMVECs). **(A)** Flow diagram of the initial strategy for presumed rat PMVEC isolation and phenotyping by immunocytochemistry and immunofluorescence. (**B)** Approach to isolating and confirming rat PMVECs acquired *ex vivo* by fluorescence activated cell sorting. The figure corresponding to data for a step in the approach is provided. HLF, human lung fibroblast; HPAEC, human pulmonary artery endothelial cell; HPASMC, human pulmonary artery smooth muscle cell; MCT, monocrotaline; RLF, rat lung fibroblast; RPAEC, rat pulmonary artery endothelial cell; RPASMC, rat pulmonary artery smooth muscle cell; rat PMVEC, rat pulmonary microvascular endothelial cell; SD, Sprague Dawley.

The overarching objective of this project was to isolate high-quality mRNA from rat PMVECs acquired *ex vivo* without tissue culturing. We chose to study rats based on severe histopathological remodeling and pulmonary hypertension that are reported for experimental PAH in this species compared to other rodent models [4]. Animals were handled in accordance with the National Institutes of Health *Guide for the Care and Use of Laboratory Animals* and all procedures were approved by the Brigham and Women’s Hospital Animal Care and Use Committee. A summary of the overall experimental workflow is provided in **Fig 1**.

We first focused on immunophenotyping cells harvested from normal rats to demonstrate endothelial cell type **(Fig 1A)**. Male Sprague Dawley rats (200-225g, Charles River) were anesthetized with ketamine (50 mg/kg)/xylazine (10 mg/kg), sacrificed by exsanguination under general anesthesia, and the outer ~4 mm of peripheral lung tissue was resected [6,12]. The whole lung segments were stored in cold Dulbecco’s Modified Eagle Medium (Life Technologies, catalog #11995–065) briefly until digestion was performed by submerging the tissue in type II collagenase (1 mg/mL, Worthington Biochemical, catalog #LS004176) for 20 min at 37°C, with 5% CO_2_ and 90% humidity.

Next, the digested lung fragments were transferred to a culture dish containing endothelial cell-specific culture medium (Vasculife with EnGS Life Factors Kit, Lifeline Cell Technology, catalog #LL-0004) supplemented with 1% (vol/vol) penicillin/streptomycin (Thermo Fisher, catalog #15140122)/amphotericin B (catalog #A2942-50ML), minced with sterile scissors (~100 times), and the tissue homogenate was filtered through a 70 μm cell strainer (Falcon, catalog #352350). The filtrate was then passed through a 20 μm strainer (Pleuriselect, catalog #43-50020-01). Trapped cells were eluted into culture medium and centrifuged at 330 x *g* for 5 min to generate a cell pellet. The pellet was resuspended and plated on a gelatin-coated P60 culture dish, incubated under standard conditions in endothelial-selective culture medium (Vasculife with EnGS Life Factors Kit, Lifeline Cell Technology) supplemented with 1% (vol./vol.) penicillin/streptomycin. Cells were grown to confluence with culture medium changes every 48 hr.

An endothelial phenotype was confirmed in confluent cells by phase contrast microscopy. These were presumed to be rat PMVECs (referred to as ‘presumed rat PMVECs’ throughout). Rat PMVECs used in ICC, IF, and pilot flow cytometry experiments were cultured (**Fig 1A**), and rat PMVECs used for RNA isolation were not cultured (**Fig 1B**).

### Determining cell type by immunocytochemistry

Immunocytochemistry (ICC) was performed on presumed rat PMVECs using the 3,3´-diaminobenzidine substrate (DAB) method, as reported by our laboratory previously, with some modifications [1]. In addition, we confirmed with the vendors that the tested antibodies were compatible with this method. Cells were fixed in ice cold acetone or methanol, blocked in 10% bovine serum albumin (BSA), and then incubated with anti-CD31 primary antibodies #1 or #2, or anti-IgG (Ab #3) as control (dilution 1:100). The secondary antibodies (Abs #4,5) were incubated at a dilution 1:500. Cells were imaged using an Olympus BX51 microscope, Retiga 3000 camera (1–5 randomly-selected fields/condition). The DAB substrate luminosity was quantified using Image J (NIH) and expressed in arbitrary units (a.u.) normalized to IgG. There was no meaningful difference in CD31 or α-SMA signal between acetone and methanol fixation (**S1 Fig**).

**Fig 2:**
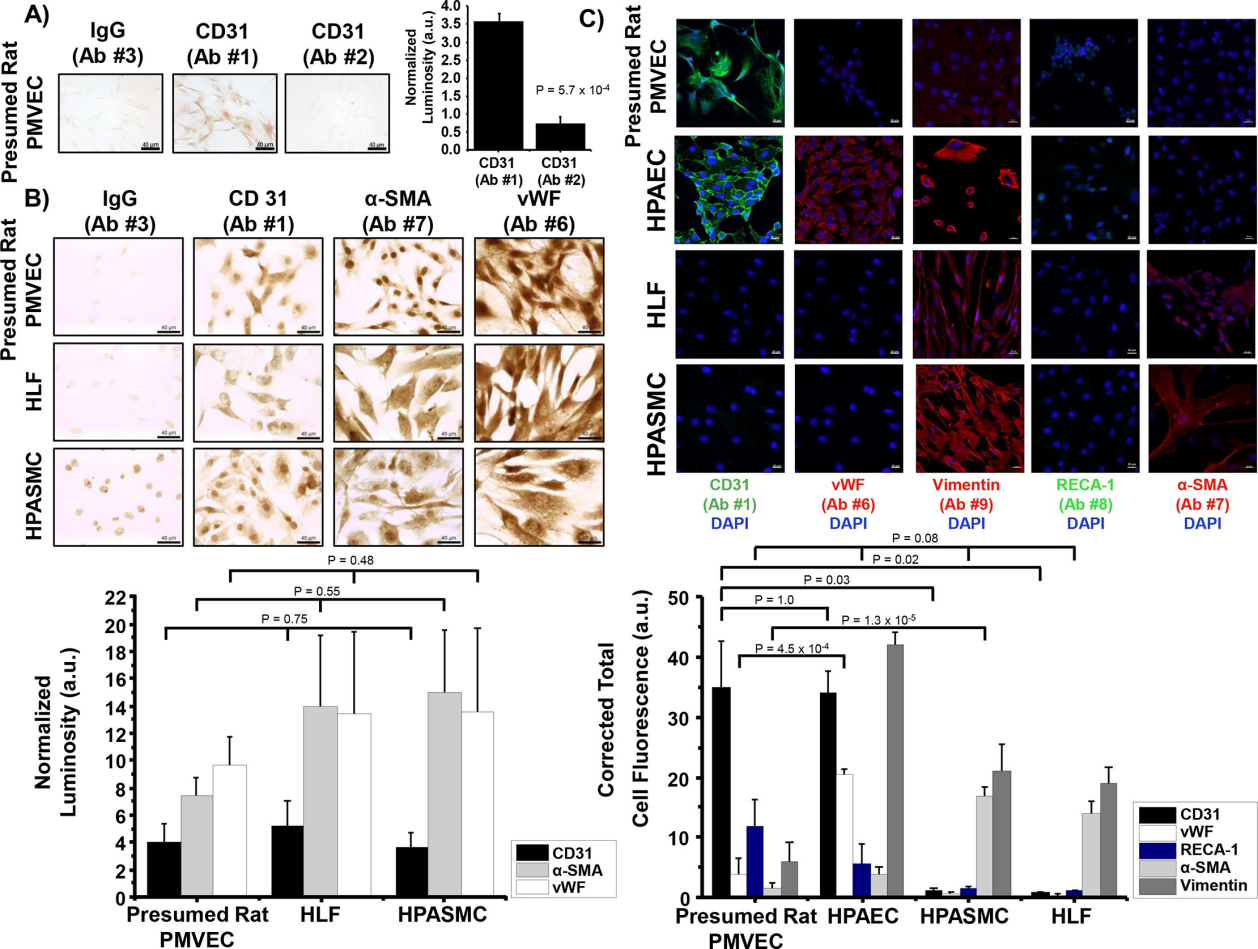
Immunocytochemistry and immunofluorescence were unsuccessful for definitively identifying presumed pulmonary microvascular endothelial cells (PMVECs) isolated from rats *ex vivo*. **(A)** Peripheral rat lung tissue underwent mechanical and enzymatic dissociation, and the cell pellet was cultured in endothelial-selective medium. Presumed rat PMVECs were analyzed using anti-CD31 Abs #1 and #2 immunocytochemistry (ICC). Luminosity was normalized to IgG control. **(B)** Anti-CD31 Ab #1, anti-von Willebrand factor (vWF) Ab #6, and anti-smooth muscle actin (α-SMA) Ab #7 ICC performed in presumed rat PMVECs, human lung fibroblasts (HLFs) and human pulmonary artery smooth muscle cells (HPASMCs) shows no significant difference in signal intensity by cell marker across different cell types. **(C)** Presumed rat PMVECs and HPAECs, HPASMCs, as well as HLFs as controls were analyzed by immunofluorescence (IF). Only CD31, and not vWF, signal was increased in presumed rat PMVECs compared to non-endothelial controls. Representative photomicrographs shown. a.u., arbitrary units. Student’s unpaired t-test or ANOVA. Means ± SE, N = 3-5/condition.

We observed that CD31 expression varied significantly between Ab #1 and Ab #2 (3.6 ± 0.2 vs. 0.7 ± 0.2 a.u., p = 5.7 x 10^−4^, N = 3/condition) (**Fig 2A**). Based on this result, we tested the specificity of anti-CD31 ICC using Ab #1 (which had the stronger signal of the two anti-CD31 Abs) in HPASMCs, HLFs, and presumed rat PMVECs. We also performed ICC using an antibody against von Willebrand factor (vWF) (Ab #6) and anti-α-smooth muscle actin (α-SMA) (Ab #7) (dilution 1:100–1:1000), which are commonly used endothelial- and smooth muscle cell-specific markers, respectively. We observed CD31 expression in presumed rat PMVECs, but it was not significantly different from HPASMCs and HLFs (4.1 ± 1.2 vs. 5.2 ± 1.9 [HPASMCs] and 3.6 ± 1.1 [HLFs] a.u., p = 0.75, N = 3-5/condition). Furthermore, anti-CD31, anti-vWF, and anti-α-SMA ICC signal detection was observed in all cell types (**Fig 2B**), suggesting that this methodology was invalid for confirming the identity of the isolated presumed rat PMVECs.

### Determining cell type by immunofluorescence

Presumed rat PMVECs as well as HPAECs and HLFs were fixed in ice-cold acetone or methanol and incubated for 60 min at room temperature in blocking solution (1% BSA, 10% goat serum, and 0.1% Tween-20 in PBS). Cells were then labeled with antibodies against CD31 (Ab #1, dilution 1:100), vWF (Ab #6, dilution 1:400), rat endothelial cell antigen-1 (RECA-1) (Ab #8, dilution 1:100), α-SMA (Ab #7, dilution 1:100) and vimentin (Ab #9, dilution 1:250). Samples were incubated with a secondary antibody conjugated to Alexa Fluor 647 or 488 (Abs #10, #11) (dilution 1:200–1:500) prior to visualization using confocal microscopy (Zeiss LSM-800, 20X Objective). Corrected total cell fluorescence was measured in a.u. using identical microscope and laser settings for each marker in 3–8 randomly-selected fields/condition. Colocalization was quantified in Zen Blue (Zeiss) by the Manders correlation coefficient using single-color controls to determine the threshold of colocalization for each fluorophore (**S2 Fig**) [29].

Similar levels of CD31 signal were observed between presumed rat PMVECs and HPAECs (34.8 ± 7.8 vs. 34.0 ± 3.8 a.u., p = 1.0, N = 6-8/condition). The CD31 level was significantly higher in presumed rat PMVECs than in HPASMCs (34.8 ± 7.8 vs. 1.3 ± 0.3 a.u., p = 0.03, N = 3-8/condition) or HLFs (34.8 ± 7.8 vs. 0.8 ± 0.3 a.u., p = 0.02, N = 3-8/condition). To support these findings, we tested vWF as a second endothelial marker. However, we observed no significant difference in vWF levels between presumed rat PMVECs and HPASMCs (3.8 ± 2.8 vs. 0.7 ± 0.2 a.u., p = 0.62, N = 3-4/condition) or HLFs (3.8 ± 2.8 vs. 0.2 ± 0.4 a.u., p = 0.50, N = 3-4/condition). Although significant vWF signal was not observed in presumed rat PMVECs relative to non-endothelial controls, vWF did appear to co-localize with CD31 compared to HPASMCs (0.71 ± 0.13 vs. 0.06 ± 0.06, Manders colocalization coefficient, p = 1.32 x 10^−4^, N = 6) and HLFs (0.71 ± 0.13 vs. 0.06 ± 0.06, Manders colocalization coefficient, p = 1.28 x 10^−4^, N = 6). In presumed rat PMVECs, methanol fixation was not associated with a change in CD31-vWF co-localization relative to acetone fixation (0.72 ± 0.08 vs. 0.71 ± 0.13, Manders colocalization coefficient, p = 0.93, N = 3) (**S2 Fig**).

Compared to CD31, RECA-1 expression was weak in presumed rat PMVECs. Furthermore, inconsistent signal was observed in experimental iterations. Indeed, a statistically significant difference in RECA-1 signal was not observed in presumed rat PMVECs compared with HPASMCs or HLFs (11.9 ± 4.5 vs. 1.6 ± 0.2 vs. 1.1 ± 0.2, a.u., p = 0.08, N = 3/condition) (**Fig 2C**).

### Cell identification by flow cytometry: Establishing a positive control

Our experiments using ICC and IF aimed to confirm the identity of cells isolated from rats *ex vivo* using two standard lineage markers for ECs, but produced conflicting results. Therefore, flow cytometry was performed next **(Fig 1B)**. To establish a positive control for the identification of pulmonary endothelial cells by flow cytometry, cultured HPAECs, HPASMCs, and HLFs were grown to confluence, lifted with Accutase (Thermo Fisher, catalog #A1110501), and blocked with 10% BSA in PBS for 15 min at 4°C. Next, cells were incubated with directly-conjugated fluorescent antibody against CD31 (Ab #12, 3 μg/mL), CD144 (Ab #13, 4.5 μg/mL), or isotype control (Ab #15, 3.0 μg/mL; Ab #14 4.5 μg/mL) for 45 min at 4°C, washed, and resuspended in 250 μL 1% BSA in PBS for immediate analysis (details on flow cytometry methods are provided in the **S1 Supporting Information**).

**Fig 3:**
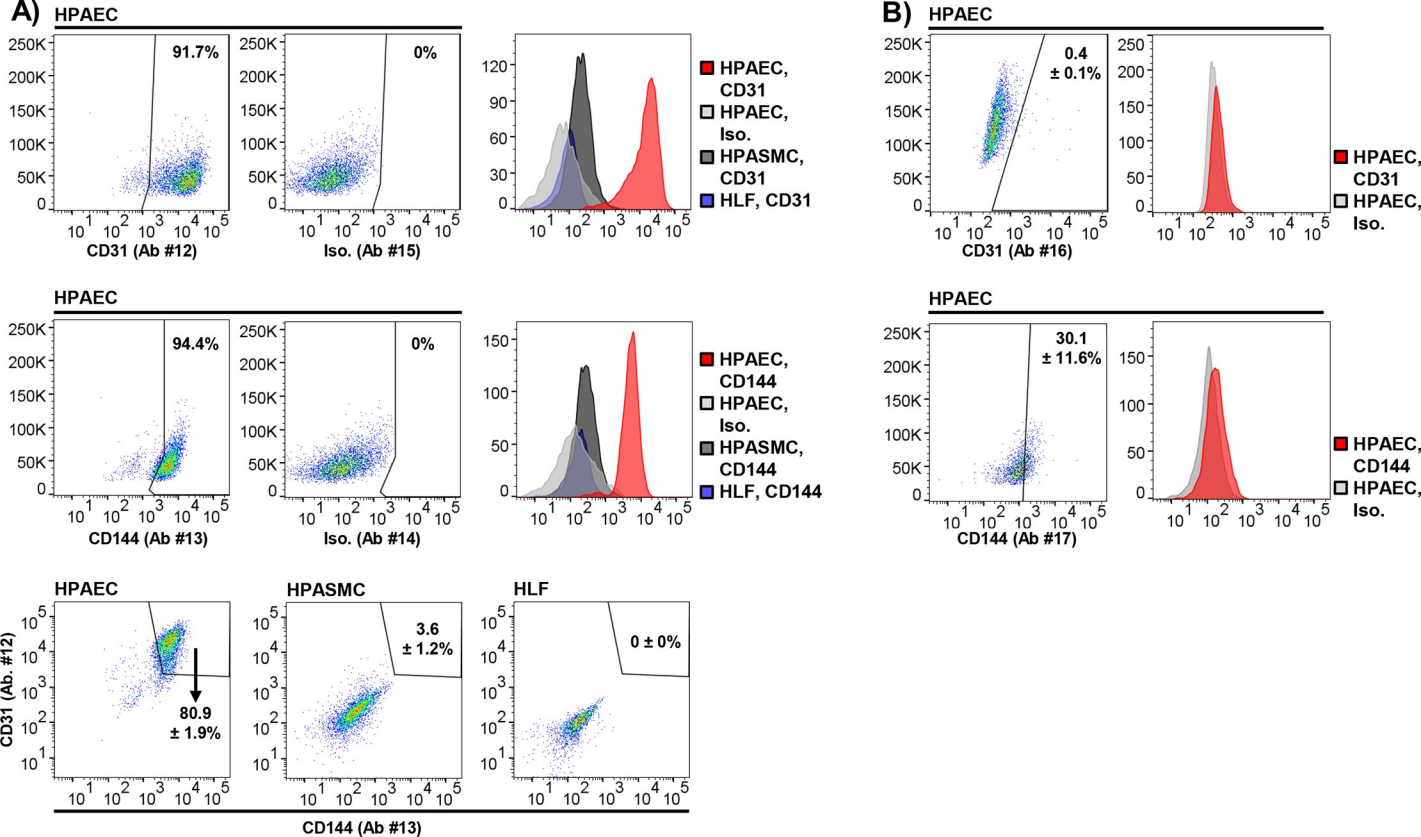
Identifying human pulmonary artery endothelial cells (HPAECs) by flow cytometry. **(A)** Commercially purchased HPAECs were analyzed by flow cytometry using anti-CD31 and anti-CD144 Abs #12 and #13. Compared with HPASMCs and HLFs, high expression of CD31 and CD144 was observed only in HPAECs. These results served as a positive control for further experiments aiming to confirm that cells isolated from rat lungs *ex vivo* were, in fact, endothelial. **(B)** Commercially purchased HPAECs were used to test the generalizability of these results. Alternative anti-CD31 and anti-CD144 antibodies did not reliably identify endothelial cell surface markers, supporting our earlier findings indicating variability in reactivity (i.e., quality) of tested antibodies across experimental methods, including flow cytometry. Representative plots and histograms shown. Means ± standard error, % CD31 or CD144 positive, N = 4-5/condition. Ab, antibody; Iso, Isotype control.

We observed double CD31 + CD144 positivity for 80.9 ± 1.9% of HPAECs compared to 0 ± 0% of HLFs (p = 2.7 x 10^−5^, N = 4/condition) and 3.6 ± 1.2% of HPASMCs (p = 3.6 x 10^−8^, N = 4/condition) (**Fig 3A**). These results were not reproduced by alternative antibodies targeting endothelial-specific epitopes: anti-CD31 Ab #16 (18 μg/mL) and anti-CD144 Ab #17 (9 μg/mL) identified marker positivity for 0.4 ± 0.1% and 30.1 ± 11.6% of HPAECs, respectively (N = 5/Ab) (isotype controls were Ab #14 and Ab #18 for CD31 and CD144, respectively) (**Fig 3B**). These findings reinforced the importance of validating specific antibodies targeting the same protein for flow cytometry to optimize results, and provided a positive control for additional flow cytometry experiments focusing on identification of presumed rat PMVECs.

### Cell identification by flow cytometry: Confirming isolated rat PMVEC identity

#### Validation of endothelial surface markers for flow cytometry

There are limited data on the use of an optimal antibody labeling strategy for the immunophenotyping of rat lung endothelial cells by flow cytometry. Therefore, we tested a range of endothelial surface marker antibodies on presumed rat PMVECs. Commercially purchased RPAECs served as a positive control.

**Fig 4:**
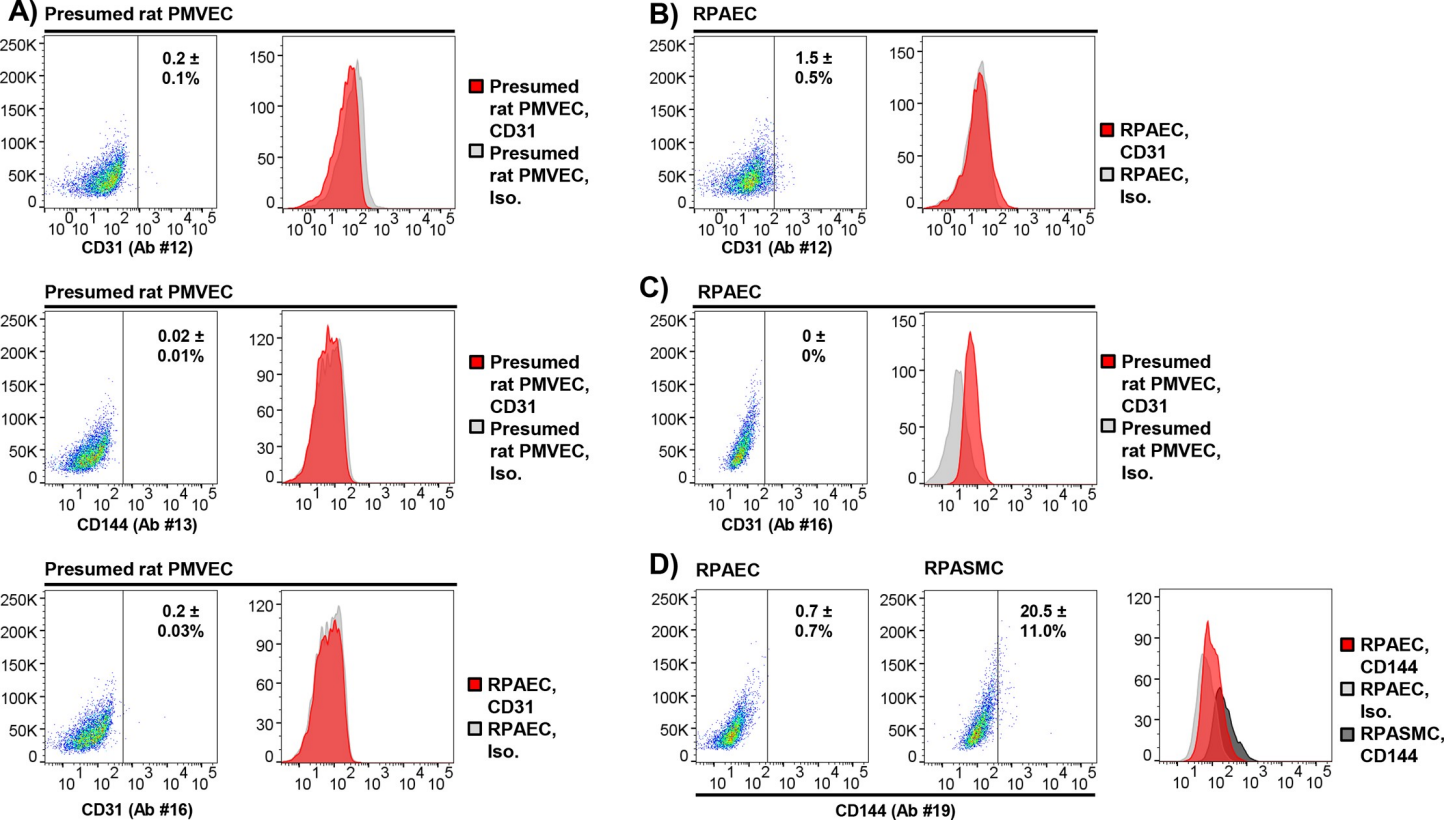
Identifying rat pulmonary endothelial cells by flow cytometry. Presumed rat PMVECs isolated by mechanical and enzymatic dissociation of peripheral lung and culture in endothelial-selective medium, commercial rat pulmonary artery endothelial cells (RPAECs), or rat pulmonary artery smooth muscle cells (RPASMCs) were labeled with antibodies against endothelial surface markers. **(A)** Anti-CD31 (Ab #12 and #16) and CD144 (Ab #13) signal was not observed in presumed rat PMVECs by flow cytometry (N = 3/condition). **(B)** Labeling of RPAECs was also not observed for anti-CD31 antibodies #12 (N = 4/condition) and **(C)** #16, respectively (N = 3/condition). **(D)** False-positive signal was detected in RPASMCs labeled with anti-CD144 Ab #19 (N = 4/condition). Representative plots and histograms shown. Means ± standard error, % CD31 or CD144 positive. Ab, antibody; Iso, Isotype control.

Presumed rat PMVECs isolated from normal rat lungs (as described in the **S1 Supporting Information**) were labelled with antibodies against CD31 (Ab #12 or #16, 18 μg/mL), CD144 (Ab #13, 18 μg/mL) or isotype control (Ab #15, Ab #14, 18 μg/mL), as these were the antibodies that effectively identified HPAECs by flow cytometry. Although rat is not listed as a target species for Ab #13 by its vendor, we nonetheless included it in this experiment because we observed wide variability in antibody reactivity across species for many antibodies. For example, Ab #12 is not recommended by the vendor to label human endothelial cells but did so effectively (**Fig 3A)**. In presumed rat PMVECs, no meaningful labeling was observed for CD31 or CD144 by Ab # 12, 16, and 13, respectively, compared to isotype control (**Fig 4A**). Similarly, Ab #12 and #16 did not result in meaningful labeling of commercial RPAECs compared to isotype control (**Figs 4B and 4C**). In the case of Ab #19, a high degree of false-positive, but not true-positive, target labeling was observed despite using a conservative blocking step to attenuate non-specific signal (10% BSA) (**Fig 4D**).

**Fig 5:**
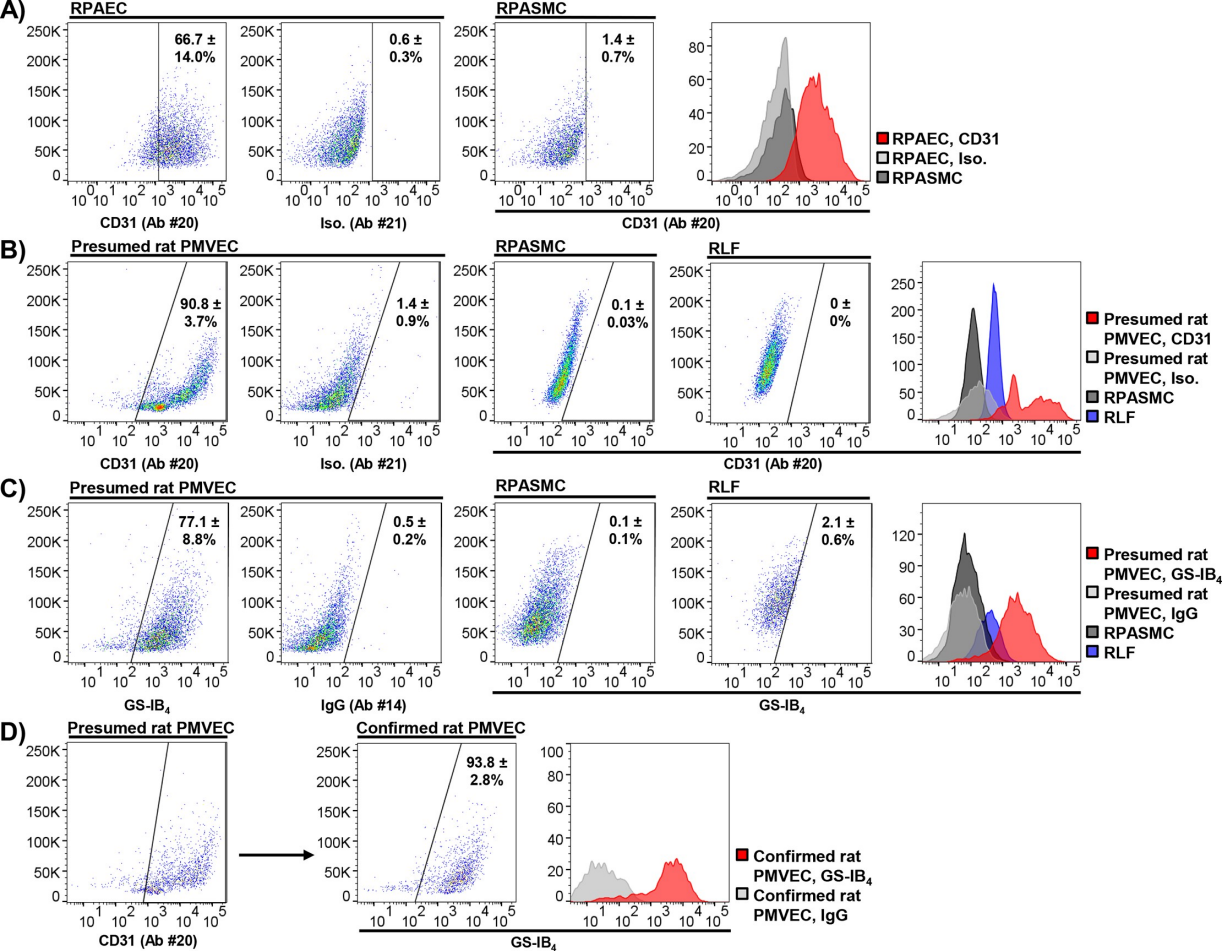
Fluorescence-activated cell sorting (FACS) permits isolation of rat pulmonary microvascular endothelial cells (PMVECs). Presumed rat PMVECs isolated from peripheral lung by immunomagnetic anti-CD31 bead selection, as well as commercial rat pulmonary artery endothelial cells (RPAECs), rat lung fibroblasts (RLFs) and RPASMCs were analyzed by flow cytometry directed against endothelial surface markers. **(A)** Anti-CD31 Ab #20 selectively labeled RPAECs relative to isotype control and RPASMCs (N = 4/condition). **(B)** Presumed rat PMVECs demonstrate specific anti-CD31 labeling relative to commercial rat lung fibroblasts (RLFs) and RPASMCs (N = 3/condition). **(C)** Presumed rat PMVECs also demonstrate specific signal for isolectin 1-B_4_ from *Griffonia simplicifolia* (GS-IB_4_) relative to RLFs and RPASMCs (N = 4/condition). **(D)** Over 90% of CD31-positive presumed rat PMVECs co-label with GS-IB_4_ (N = 3/condition). Confirmed rat PMVECs were defined as those cells positive for both CD31 and GS-IB_4_ by flow cytometry. Representative plots and histograms shown. Means, % CD31 or GS-IB_4_ positive. Ab, antibody; Iso, Isotype control.

Ultimately, it was determined that anti-CD31 Ab #20 (R&D Biosystems, catalog #FAB3628P) labeled commercial RPAECs effectively and selectively relative to isotype control (Ab #21, R & D Biosystems, catalog #IC108P) (66.7 ± 14.0 vs. 0.6 ± 0.3, % CD31 positive, p = 0.003, N = 4/condition, 20 μg/mL) or RPAMSCs (66.7 ± 14.0 vs. 1.4 ± 0.7, % CD31 positive, p = 0.003, N = 4/condition, 20 μg/mL) (**Fig 5A)**.

#### Magnetic bead-based cell isolation

Based on our findings demonstrating that anti-CD31 Ab #20 labelled commercially purchased RPAECs successfully, we next utilized this antibody to isolate presumed normal rat PMVECs using coated magnetic beads. Importantly, this approach is not contingent on population expansion *in vitro*, which, in turn, is associated with a shift in the molecular phenotype of cells [20]. A total of 100 μL of resuspended magnetic bead solution (Cellection Pan Mouse IgG Kit, Thermo Fisher, catalog #11531D) was incubated with 17 μg/ml anti-CD31 antibody (Ab #22) and washed in 0.1% BSA in PBS per the manufacturer directions and according to methods detailed in the **S1 Supporting Information**.

#### FACS preparation

The method for FACS preparation is outlined in the **S1 Supporting Information**. Presumed rat PMVECs demonstrated strong positivity for CD31 relative to isotype control (90.8 ± 3.7 vs. 1.4 ± 0.9, % positive for CD31, p = 1.9 x 10^−5^, N = 3/condition) (**Fig 5B**). By contrast, no significant CD31 labeling was observed in commercial RPASMCs (90.8 ± 3.7 vs. 0.1 ± 0.03, % positive for CD31, p = 1.6 x 10^−5^, N = 3/condition) and RLFs (90.8 ± 3.7 vs. 0 ± 0, % positive for CD31, p = 0.002, N = 3/condition) (**Fig 5B**)

Given the challenges associated with poor antibody precision, we co-labeled cells with GS-IB_4_ as a strategy to enhance the specificity of our cell yield. Specifically, GS-IB_4_ binds α-D-galactosyl residues, and has been shown previously to preferentially label endothelial cells of microvascular origin using IF and flow cytometry [5–8,30,31]. Presumed rat PMVECs were blocked as described previously and incubated with 20 μg/mL anti-CD31 antibody #20, 5 μg/mL GS IB_4_, or control (antibodies #21 or #15, respectively) for 45 min at 4°C in a total of volume of 50 μL. Compared to IgG control Ab #14, GS-IB_4_ signal was strongly positive in presumed rat PMVECs (0.5 ± 0.2 vs. 77.1 ± 8.8, % GS IB_4_ positive, p = 9.3 x 10^−4^, N = 3/condition). Compared to presumed rat PMVECs, we observed no meaningful GS-IB_4_ labeling in commercial RPASMCs (77.1 ± 8.8 vs. 0.1 ± 0.1, % GS-IB_4_ positive, p = 9.2 x 10^−4^, N = 3/condition, 5 μg/mL) and RLFs (77.1 ± 8.8 vs. 2.1 ± 0.6, % GS-IB_4_ positive, p = 0.001, N = 3/condition, 5 μg/mL) (**Fig 5C)**. Furthermore, 93.8 ± 2.8% of CD31-positive cells also labeled positively for GS-IB_4_ (5 μg/mL, N = 3/condition) (**Fig 5D**). By propidium iodide analysis, GS-IB_4_ labeling did not adversely influence the viability of presumed rat PMVECs (86.6 ± 7.7 vs. 86.9 ± 6.9, % viable cells, p = 0.98, N = 3/condition).

#### Preparing rat PMVECs isolated by FACS for transcriptomic analyses

Overall, CD31-positive cells comprised 86.7 ± 2.9% (N = 3) of the total cell population isolated from rat lungs by magnetic bead selection. Although this is consistent with published data on other endothelial cell types, it is possible that including cells that were not positive for CD31 could adversely influence results of subsequent transcriptomic analyses [16]. Thus, we confirmed rat PMVEC identity by virtue of CD31 + GS-IB_4_ double-positivity sorted by FACS. Only that cell population was used for isolating RNA in preparation for transcriptomic analyses. The FACSAria Special Order flow cytometer was used for cell sorting, as detailed in the **S1 Supporting Information**.

To demonstrate that immunomagnetic bead selection for CD31 followed by FACS isolation of CD31 + GS-IB_4_ double-positive cells is a valid method of isolating high-quality RNA for transcriptomic profiling in experimental PAH, male Sprague Dawley rats were administered a single intraperitoneal injection of monocrotaline (MCT) (Sigma C2401-1G) or normal saline control on day 0 of the protocol. Confirmed rat PMVECs comprised 78.1 ± 5.8% and 61 ± 7.3% of viable presumed PMVECs, in control and MCT-PAH, respectively (p = 0.1) (**S3 Fig**). RNA was isolated from confirmed MCT-PAH PMVECs on day 23 and assayed for quality (Agilent 2100 Bioanalyzer). RNA integrity number (RIN) (Agilent) was 9.2 ± 0.1 vs. 8.7 ± 0.1 (p = 0.003) for control vs. MCT-PAH, respectively (N = 6 rats/condition) (**Table 2**). Full RNA electropherograms are available for all samples in **S2 Supporting Information**.

**Table 2:** Assessment of RNA quality by RNA integrity number.

RNA Integrity Number (Control)	RNA Integrity Number (MCT-PAH)
9.2	8.9
9.2	8.8
8.9	8.8
9.7	8.3
9.1	8.5
9.1	8.6

Confirmed PMVEC RNA was isolated from monocrotaline (MCT) and control-treated rats, and the RNA Integrity Number (RIN) was determined on an Agilent 2100 Bioanalyzer. PAH, pulmonary arterial hypertension.

## Discussion

In this report, we detail a novel approach to isolating PMVECs directly from rat lungs using flow cytometry. Specifically, magnetic bead selection for CD31 followed by FACS identified a high rate of double positive cells for CD31 and GS-IB_4_, which enhanced the specificity of cell recovery and permitted isolation of high-quality RNA from PMVECs without culturing cells. This allows for next generation transcriptomic analyses on PMVECs *ex vivo* without passaging, which is associated with changes in genomic and proteomic programing that may limit translational relevance of results [20]. Our data also illustrate discrepancies in the quality of commercially available and commonly referenced biomaterials that are used to identify cell type. Thus, a second major finding of this work relates to the critical importance of investigator-initiated validation of reagents used in PAH and other experimental biology fields.

Studying PAECs isolated from patients is ideal for experimental research, but PAH is a rare disease, limiting donor availability from explanted lungs, and accessing distal pulmonary arterials using minimally invasive methods is associated with risk. Isolating and phenotyping primary human or murine pulmonary endothelial cells has been reported previously using serial passaging with endothelial cell-selective media, anti-endothelial antibody-coated magnetic beads, or FACS [5–7,9–12,15,17,19]. However, experimental murine models do not recapitulate many features of PAH observed in patients, particularly plexogenic vascular lesions or severe pulmonary hypertension. By contrast, there are few reports focusing on methods for isolating pulmonary artery endothelial cells in rats despite important advantages of PAH models in this species. This may partly reflect inconsistent quality for rat-compatible commercially available anti-endothelial antibodies. For example, the anti-CD31 monoclonal antibody clone TLD-3A12 is commonly referenced as a rat lung endothelial marker [10,19,32,33]. But in our experience, this clone was ineffective for profiling presumed PMVECs by ICC or flow cytometry.

Our findings show that Ab #12, which is also derived from clone TLD-3A12, had excellent sensitivity and specificity for the detection of CD31 on HPAECs by flow cytometry. Importantly, *Homo sapiens* is not listed as a target species by the antibody vendor. This observation is consistent with limited access to (or availability of) data on the target antigen or derivative epitope for that clone specifically, as well as other antibodies more generally, which ultimately confounds predicting relevant biophysical interactions that may explain experimental results. Other antibodies against endothelial surface antigens were associated with poor specificity. For example, in some experiments we observed strong CD31 and vWF expression in HPASMCs. This trend was not limited to ICC or IF analytical methods: a false detection rate for CD144 positive cells was observed in >20% RPASMCs analyzed by flow cytometry, which is consistent with published reports from others [5].

**Fig 6:**
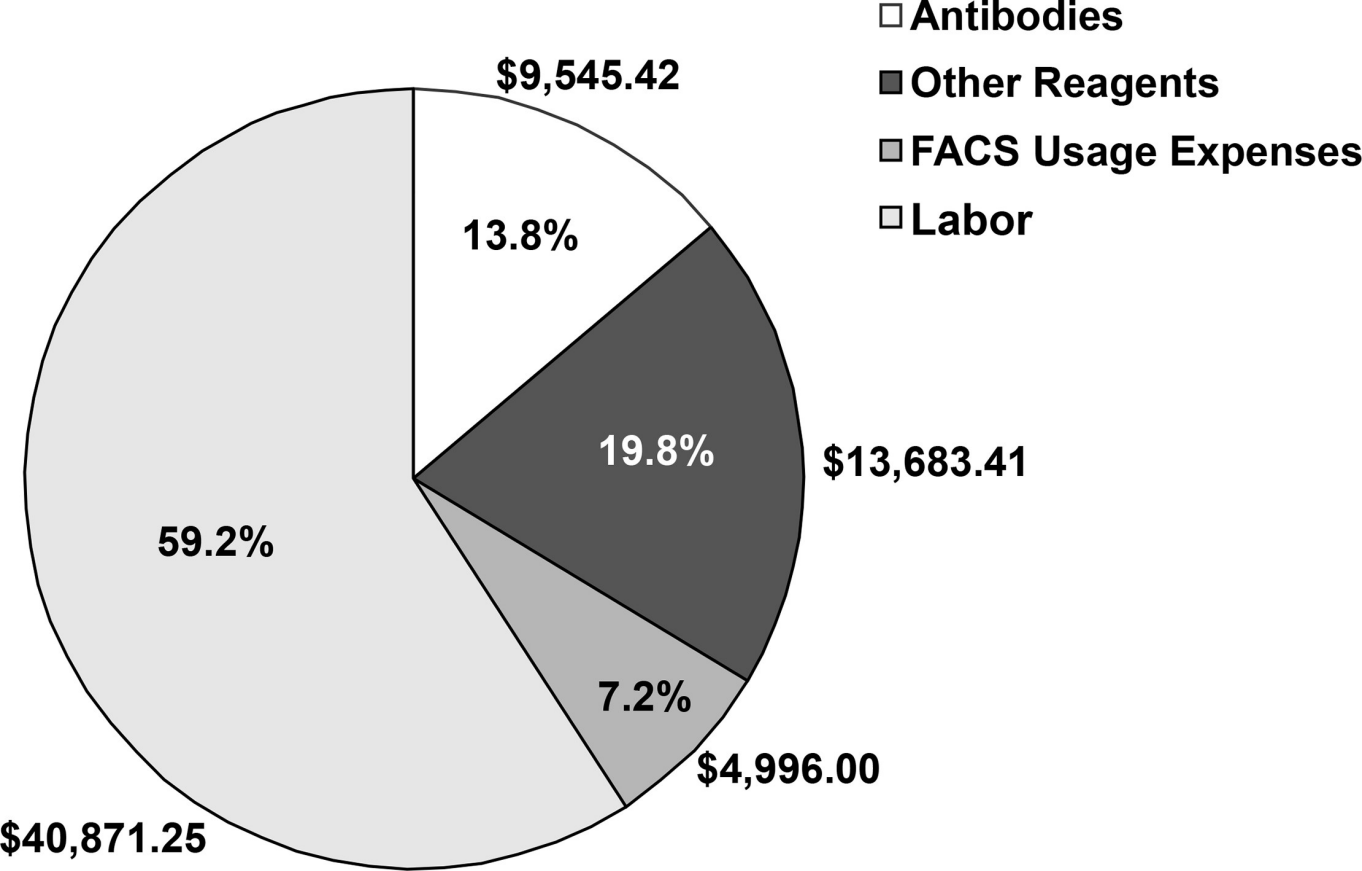
Reagent, personnel, and equipment costs attributable to the validation of commercial biomaterials. Distribution of expenses attributable to the validation of commercial products used in the isolation of rat pulmonary microvascular endothelial cells. FACS, fluorescence-activated cell sorting.

Taken together, these data suggest that the recognition of pulmonary endothelial antigens by commercially available antibodies is variable and assay-dependent. This experience is in concert with accumulating data implicating reagent quality in the synthesis of low fidelity or irreproducible findings [24]. Our data expand this field by cataloguing the pervasiveness and extent of this problem across a wide spectrum of methodologies and antibodies that are purported to share the same target. Although these findings clarify the importance of using valid reagents for fundamental experimentation (i.e., cell type identification), a simple solution to this problem appears less certain. Antibody validation imposes a substantial financial and time burden on scientific investigators. For example, over the course of this project, antibody validation experiments consumed an estimated $69,096 in reagent, personnel, and FACS expenses—$9,545 (13.8%) of which was spent on the purchase of commercial antibodies (**Fig 6)**. Reagent validation may, thus, warrant greater consideration when considering laboratory budgets by investigator and sponsor alike.

The intent of this project was to isolate high-quality mRNA from rat PMVECs successfully, and, therefore, an exhaustive evaluation of all commercially available products and labeling conditions was not performed. For example, antibodies against intracellular antigens were not tested since this necessitates cell fixation and/or permeabilization for FACS. However, others have reported that efficient RNA recovery from fixed cells may be feasible [34–36]. Although most cells and biomaterials were fresh, our experiments did not control for age and variability in storage conditions, which may have affected our results. We recognize that antibody labeling experiments typically require optimization; it is possible that different extracellular matrix coatings *in vitro*, cell culture conditions, and methods of fixation or immunolabeling could influence the results of the IHC, IF, and flow cytometry analyses. Significant RECA-1 expression was not observed by IF in presumed rat PMVECs. The mechanism of this observation was not explored, as we ultimately favored a FACS-based rat PMVEC isolation strategy that was not contingent on RECA-1 labeling. In general, we followed vendor-recommended antibody labeling protocols, and performed additional attempts at optimization when possible. Budgetary and time constraints precluded an independent evaluation of the purity of each lot of commercial primary cells,[37] although each primary cell type was analyzed by flow cytometry.

Further experiments are needed to confirm that our methodology isolated PAECs exclusively. Others have reported CD31 and α-D-galactosyl surface residue presence in non-endothelial cells, and distinguishing endothelial cells of vascular versus lymphatic origin was not a specific focus of this study [16,38–40]. However, it has been shown previously that GS-IB_4_ does not co-localize with lymphatic endothelial-specific cells (defined by LYVE-1 expression) in the lung, providing indirect evidence to cell endothelial populations isolated in our report are predominately vascular in origin [41,42]. Additionally, we used flow cytometry gating settings that were conservative, and focused on populations with the highest CD31 and GS-IB_4_ signals to minimize the chance for detection of other cell types [16,38]. Nonetheless, alternative proteins, such as endoglin and vascular endothelial growth factor receptor-2, may provide enhanced endothelial specificity and should be considered in future research.

In summary, we present a methodology for isolating PMVECs from rats *ex vivo* using flow cytometry that does not require cell culture or passaging prior to transcriptomic analysis. We identified poor sensitivity and specificity of commercially available antibodies for pulmonary endothelial antigens. These collective findings have important implications for future work in experimental PAH, particularly translational endeavors that involve interrogating the ‘omic’ profile for PMVECs, and underscores the need for investigator-driven validation of key immunological reagents.

## Supporting information

S1 Supporting Information This document contains details regarding flow cytometry setup, endothelial cell isolation methods, as well as sources and characteristics of commercial primary cells. (DOCX)

S2 Supporting Information Raw electropherograms for rat PMVEC RNA isolated from control and monocrotaline-PAH animals are provided in this document. (DOCX)

S1 Fig Detection of CD31 and α-smooth muscle actin by immunocytochemistry was similar with acetone or methanol cell fixation. Peripheral rat lung tissue was treated with mechanical and enzymatic dissociation, and the cell pellet was cultured in endothelial-selective medium. Presumed rat PMVECs were fixed in acetone or methanol and analyzed using anti-CD31 Ab #1 and anti-α-smooth muscle actin Ab # 7 immunocytochemistry. Luminosity was normalized to IgG (Ab #3). Representative images shown. a.u., arbitrary units. Student’s unpaired t-test. Means ± SE, N = 3/condition.
(TIF)

S2 Fig CD31 and von Willebrand Factor colocalize in human pulmonary arterial endothelial cells and presumed rat pulmonary microvascular endothelial cells. **(A)** Human pulmonary artery endothelial cells were labeled with either anti-CD31 Ab #1 or anti-von Willebrand Factor Ab #6 and analyzed using confocal microscopy to determine colocalization thresholds. (**B)** Peripheral rat lung tissue was subjected to mechanical and enzymatic dissociation, and the cell pellet was cultured in endothelial-selective medium. Presumed rat PMVECs, human pulmonary artery endothelial cells, human pulmonary artery smooth muscle cells, and human lung fibroblasts were fixed in acetone and co-labeled with anti-CD31 Ab #1 and anti-von Willebrand Factor Ab #6 and colocalization was measured using the thresholds established in panel (A). To enhance visualization, regions of colocalization are emphasized using a false-colored yellow overlay. **(C)** Meaningful differences in CD31-vWF colocalization were not observed between methanol and acetone fixation of presumed rat PMVECs. Representative images and scatterplots shown. AF 488, Alexa Fluor 488; AF 647, Alexa Fluor 647. Student’s unpaired t-test. Means ± SE, N = 3/condition.
(TIF)

S3 Fig Detailed gating strategy for the identification of confirmed rat pulmonary microvascular endothelial cells by CD31 and *Griffonia simplicifolia* isolectin 1-B_4_ flow cytometry. Presumed rat PMVECs were isolated without cell culture by mechanical and enzymatic digestion and immunomagnetic bead selection for CD31. Presumed rat PMVECs were labeled with anti-CD31 Ab #20 (conjugated to phycoerythrin) and *Griffonia simplicifolia* isolectin 1-B_4_ (conjugated to Alexa Fluor 488) and analyzed by flow cytometry. Fluorescence minus one controls were used to establish gates. Isotype or IgG control confirmed the specificity of cell labeling by *Griffonia simplicifolia* isolectin 1-B_4._ Viability was assessed by propidium iodide. Representative plots shown. AF 488, Alexa Fluor 488; AF 647, Alexa Fluor 647; FSC-H, forward scatter-height; PE, phycoerythrin; PI, propidium iodide; SSc-A, side scatter-area.
(TIF)

## Abbreviations

a.u.: arbitrary units
Ab: antibody
AF 488: Alexa Fluor 488
AF 647: Alexa Fluor 647
DAB, 3: 3´-diaminobenzidine
GS-IB_4_: isolectin 1-B_4_ from *Griffonia simplicifolia*
HLF: human lung fibroblast
HPAEC: human pulmonary artery endothelial cell
HPASMC: human pulmonary artery smooth muscle cell
ICC: immunocytochemistry
IF: immunofluorescence
PAH: pulmonary arterial hypertension
PE: phycoerythrin
PMVEC: rat pulmonary microvascular endothelial cell
MCT: monocrotaline
RECA-1: rat endothelial cell antigen-1
RIN: RNA integrity number
RLF: rat lung fibroblast
RPAEC: rat pulmonary artery endothelial cell
RPASMC: rat pulmonary artery smooth muscle cell
S.E.: standard error
vWF: von Willebrand factor
α-SMA: α-smooth muscle actin
