# Supplementary material for: Isolating pulmonary microvascular endothelial cells ex vivo: Implications for pulmonary arterial hypertension, and a caution on the use of commercial biomaterials
Source: PLoS One. 2019 Feb 27;14(2):e0211909. doi: 10.1371/journal.pone.0211909 (PMC6392245; doi:10.1371/journal.pone.0211909)
Supplement: S1 Supporting Information — (DOCX) [file pone.0211909.s001.docx]

**Isolating pulmonary microvascular endothelial cells *ex vivo*: implications for pulmonary arterial hypertension, and a caution on the use of commercial biomaterials**

**S1 Supporting Information**

**Detailed Methods**

**General flow cytometry setup**

Samples were analyzed on a FACS Aria Special Order or FACS Aria Fusion Flow Cytometer (Becton Dickinson), and FACSDiva Software (Becton Dickinson, Version 6.1.3) was used to determine multi-color compensation settings [1]. Fluorophores used in FACS experiments included phycoerythrin, Alexa Fluor 488, and allophycocyanin. Non-viable cells, defined as non-intact cells, were quantified by propidium iodide staining (5 mg/mL, Sigma, catalog #P4864-10ML) and excluded from the analysis. Gating was performed using fluorescence minus one controls, as published previously [1].

**Magnetic bead-based endothelial cell isolation**

Animals were anesthetized, sacrificed by exsanguination under general anesthesia, and the pulmonary artery was perfused with 4-8 mL PBS until the lungs were cleared of blood. Lungs were removed *en bloc*, and the peripheral 2-4 mm aspect of each lobe was excised (0.5-0.8 g total tissue), rinsed in PBS, dissociated (Miltenyi GentleMACS Dissociator, catalog #130-093-235, device setting: Program m_lung_01_02), enzymatically digested (Miltenyi Mouse Lung Dissociation Kit, catalog #130-095-927) at 37°C for 30 min on a rotator at 40 rpm, and dissociated again using a more vigorous program on the GentleMACS device (Device setting: Program m_lung_02_01).

The resulting tissue suspension was passed through a 70 μm cell strainer (Miltenyi, catalog #130-098-462), rinsed with 2.5 mL Buffer S (Miltenyi Mouse Lung Dissociation Kit, catalog # 130-095-927), and the filtrate was centrifuged at 2000 rpm for 5 min. The cell pellet was resuspended in 1 mL of 0.5% BSA and 2 mM EDTA in PBS in a 1.5 mL microcentrifuge tube and 100 μL of CD31-coated magnetic beads were added and incubated at 4°C for 20 min on a rotator (20 rpm). Next, the microcentrifuge tube was mounted on a magnetic particle concentrator (MPC). The supernatant was discarded, the beads were washed three times with 1 mL 0.1% BSA in PBS and then resuspended in 200 μL of room temperature culture medium (Vasculife with EnGS Life Factors Kit, Lifeline Cell Technology). To dissociate the cells from the magnetic beads, 12 μL of release buffer containing DNAse (Cellection Pan Mouse IgG Kit, Thermo Fisher) was added to the microcentrifuge tube and incubated for 15 min at room temperature on a rotator (20 rpm). The solution was pipetted up and down 10-20 times to maximize cell release and mounted on a MPC. The supernatant (which contains cells) was transferred to a new 1.5 mL microcentrifuge tube that had been pre-rinsed with culture medium. The bead fraction was resuspended in 200 μL of room temperature culture medium, mounted on the MPC, and the supernatant was recollected to maximize cell recovery.

**Flow cytometry analysis of presumed rat PMVECs isolated by bead-based method**

The cell suspension was centrifuged at 2000 rpm for 5 min at 4°C, and the pellet was resuspended in 10% BSA in PBS. Samples were blocked for 15 min at 4°C on a rocker, resuspended in 1% BSA in PBS, and labeled with 20 μg/mL anti-CD31 Ab #20 or isotype control Ab #21 in a total volume of 50 μL. Cells were incubated with antibody for 45 min at 4°C on a rocker. After incubation, labeled cells were washed in 1 mL 1% BSA in PBS to remove unbound antibody, and centrifuged at 2000 rpm for 5 min at 4°C to generate a cell pellet. The pellet was resuspended in 250 μL 1% BSA in PBS, filtered through a 35 μm nylon mesh filter, and unfixed cells were analyzed by flow cytometry as described above.

**Fluorescence activated cell sorting of rat PMVECs for transcriptomic analysis**

Sorting took place on the FACSAria Special Order flow cytometer using a 70 μm nozzle, 70 psi sheath pressure, flow rate of 2.0, and preservative-free sheath buffer (BioSure, catalog # 1020) after the system had been flushed with 10% bleach. Cells expressing the highest signals for both CD31 and GS-IB4 (i.e., confirmed rat PMVECs) were sorted into cold Trizol LS (Thermo Fisher, catalog #10296010), per the manufacturer’s recommendations. Samples were vortexed and transported on ice for immediate RNA extraction using a modification of the RNEasy column method (Qiagen, catalog #74106) [2]. For every 1 mL of Trizol LS used, 0.2 mL of chloroform (Sigma, catalog #439142-4L) was added, and samples were shaken by hand for 15 s, incubated for 3 min at room temperature, and centrifuged at 12,000 x *g* for 5 min at 4°C. The aqueous (top) phase was removed, mixed with 100% ethanol in a 1:1 ratio by volume, and 0.7 mL of the solution was then applied to an RNeasy column and centrifuged at 8000 x *g* for 15 s at 4°C. The flow-through was discarded and this process was repeated until the entire volume was applied to the column. Next, 0.7 mL of buffer RW1 was applied to the column, and it was centrifuged at 8000 x *g* for 15 s at 4°C. The flow-through was discarded, 0.5 mL of buffer RPE was applied to the column, and it was centrifuged at 8000 x *g* for 15 s at 4°C. 0.5 mL of buffer RPE was reapplied and the column was centrifuged again. The column was placed in a new 2 mL collection tube and centrifuged at 8000 x *g* for 15 s at 4°C to remove residual buffer. Finally, the column was placed in a 1.5 mL microcentrifuge tube, 40 μL of RNAse-free water was applied, and the column was centrifuged at 8000 x *g* for 15 s at 4°C to elute the RNA. Isolated rat PMVEC RNA was quantified using a Qubit 3 Fluorometer (Thermo Fisher) and RNA integrity was assessed on an Agilent Bioanalyzer.

| **Primary Cell Type** | **Vendor** | **Donor Demographics** | **Lot Number** | **Medium**  (Catalog #) |
| --- | --- | --- | --- | --- |
| RPAEC | Cell Biologics | Sprague Dawley rat | 062014110613W10 | Complete Rat Endothelial Cell Medium  (M1266) |
| RPASMC | Cell Biologics | Sprague Dawley rat | F081416W16 | Complete Smooth Muscle Cell Medium (M2268) |
| RLF | Cell Biologics | Sprague Dawley rat | 031015W14 | Complete Fibroblast Medium (M2267) |
| HPAEC | Lonza | 65 year-old male  34 year-old female  64 year-old male | 0000598033  0000657513  0000647487 | Endothelial Cell Basal Medium-2  (CC-3156) |
| HPASMC | Lonza | 57 year-old male  34 year-old female | 0000466718  0000550178 | Smooth Muscle Cell Basal Medium (CC-3181) |
| HLF | Lonza | 57 year-old female  3 year-old male | 0000543644  0000511473 | Fibroblast Basal Medium (CC-3131) |

**S1 Table**

**S1 Table**: **Source and characteristics of commercial primary cells.** Primary cells were obtained from commercial suppliers and cultured in vendor-recommended medium and conditions. HLF, human lung fibroblast; HPAEC, human pulmonary artery endothelial cell; HPASMC, human pulmonary artery smooth muscle cell; RLF, rat lung fibroblast; RPAEC, rat pulmonary artery endothelial cell; RPASMC, rat pulmonary artery smooth muscle cell.

**Supplemental References**

1. Cossarizza A, Chang H-D, Radbruch A, Akdis M, Andrä I, Annunziato F, et al. Guidelines for the use of flow cytometry and cell sorting in immunological studies. Eur J Immunol. 2017;47: 1584–1797. doi:10.1002/eji.201646632

2. Peter, White. RNA Extraction from Mammalian Tissues [Internet]. University of Pennsylvania School of Medicine, Functional Genomics Core; 2007. [Accessed 4 March 2018]. Available: https://www.med.upenn.edu/idom/derc/cores/fgc/documents/RNAExtraction-FunctionalGenomicsCoreStandardMethods.pdf
