# Supplementary material for: Isolating pulmonary microvascular endothelial cells ex vivo: Implications for pulmonary arterial hypertension, and a caution on the use of commercial biomaterials
Source: PLoS One. 2019 Feb 27;14(2):e0211909. doi: 10.1371/journal.pone.0211909 (PMC6392245; doi:10.1371/journal.pone.0211909)

**Isolating pulmonary microvascular endothelial cells *ex vivo*: implications for pulmonary arterial hypertension, and a caution on the use of commercial biomaterials**

**S2 Supporting Information**

**RNA Quality Assessment**

**Assessment of RNA Quality**

RNA was isolated from confirmed control and monocrotaline-PAH rat PMVECs, as detailed in the methods and **S1 Supporting Information**. Raw electropherograms from the Agilent 2100 Bioanalyzer used to calculate the RNA integrity number (RIN) are provided:

Control Sample 1 (RIN 9.2)


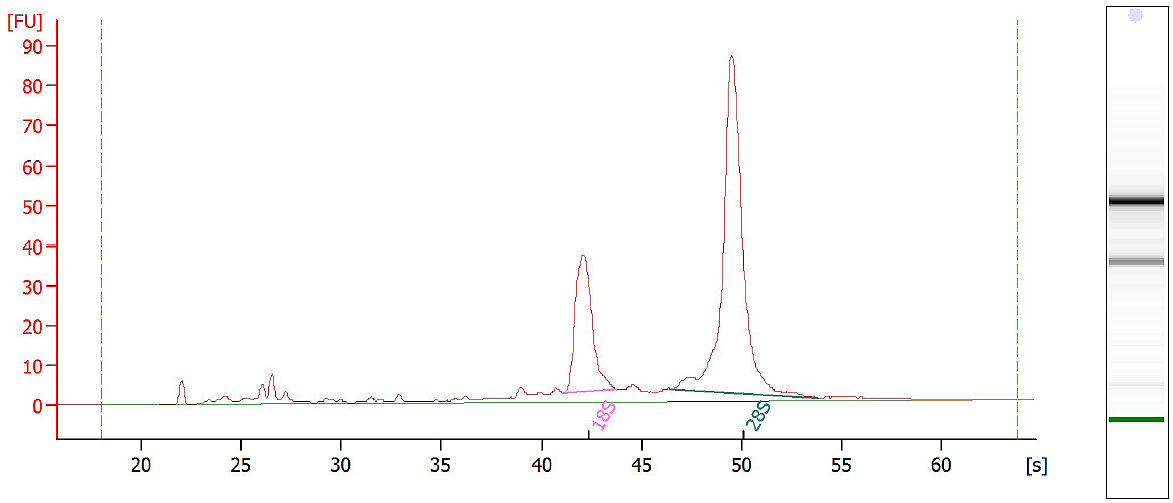


Control Sample 2 (RIN 9.2)


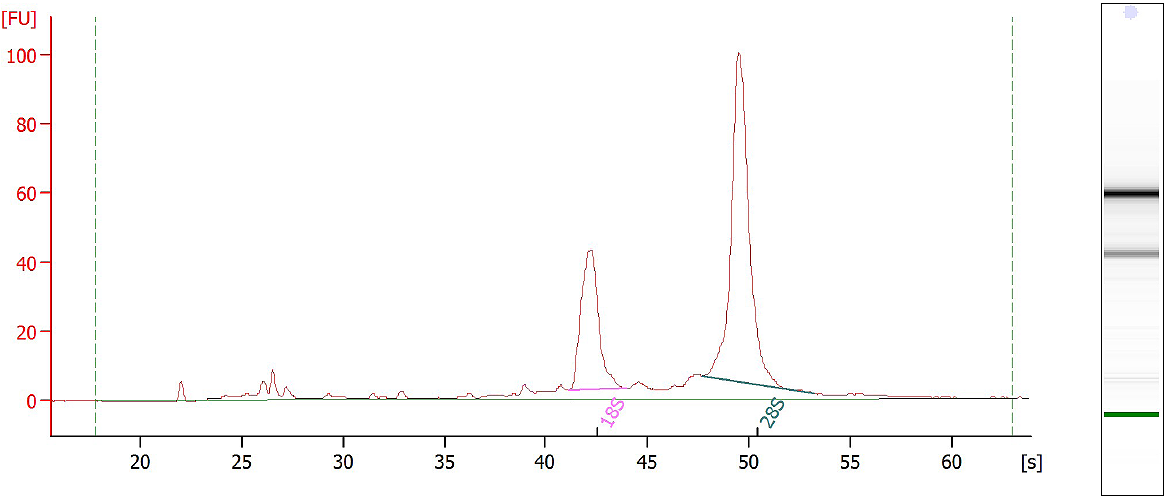


Control Sample 3 (RIN 8.9)


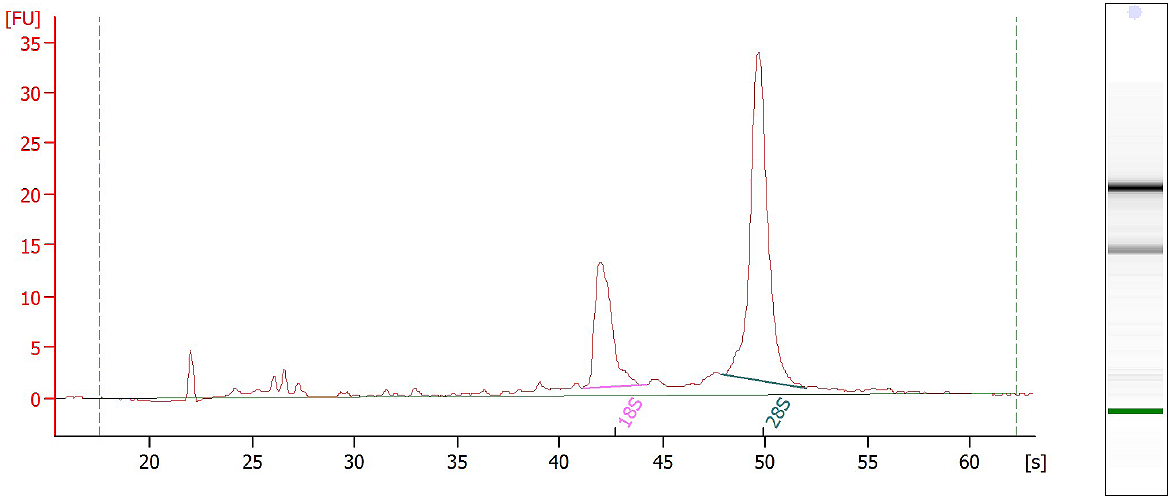


Control Sample 4 (RIN 9.7)


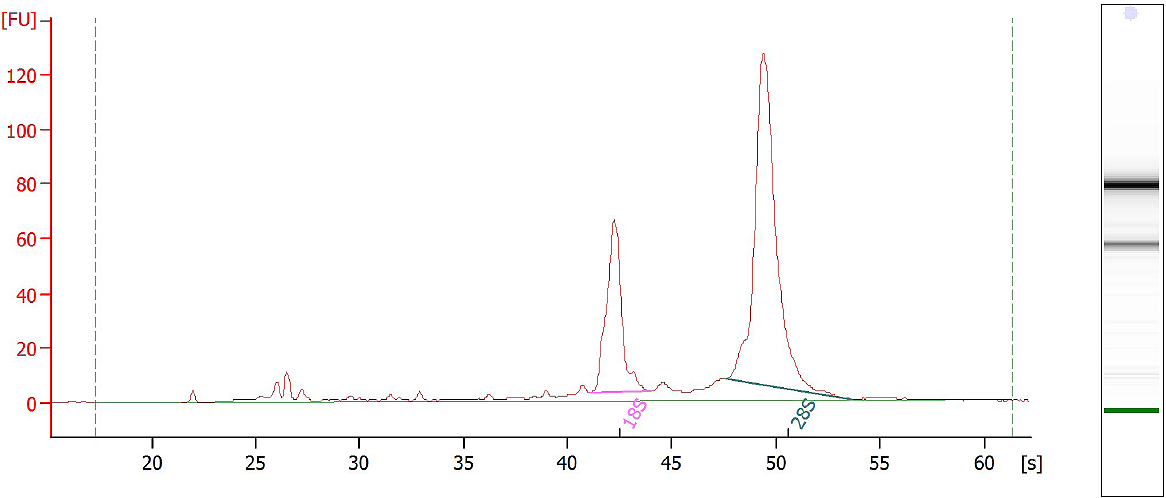


Control Sample 5 (RIN 9.1)


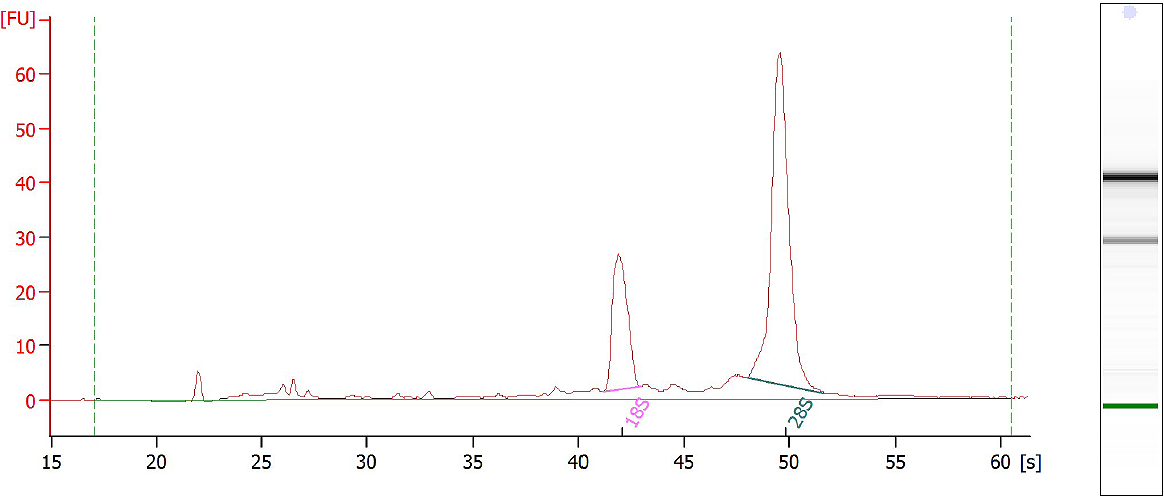


Control Sample 6 (RIN 9.1)


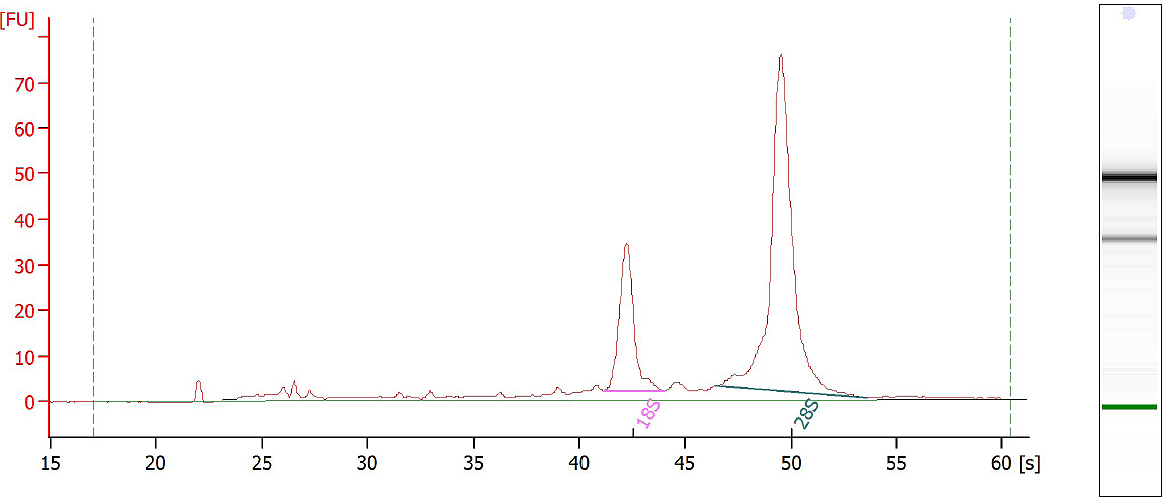


PAH Sample 1 (RIN 8.9)


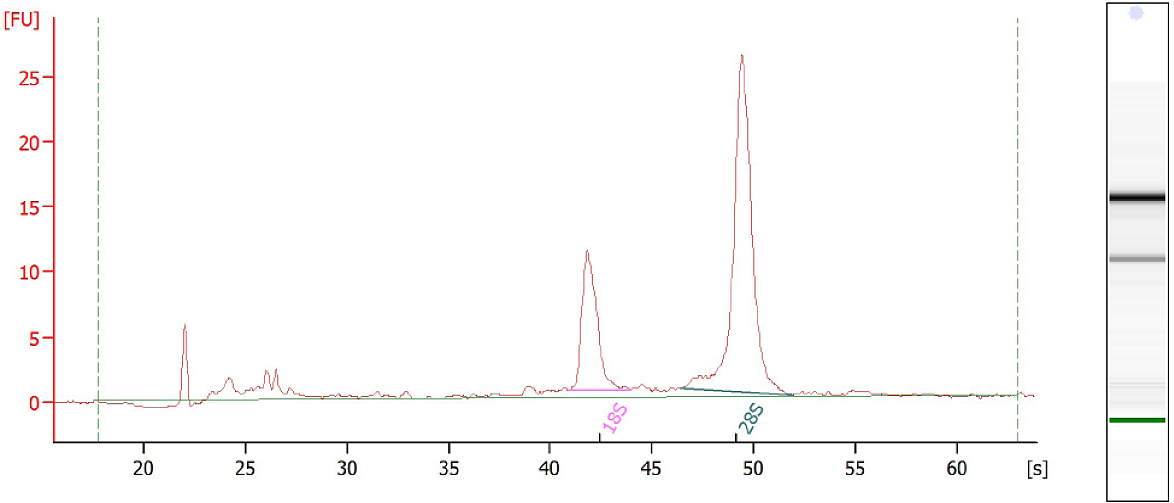


PAH Sample 2 (RIN 8.8)

PAH Sample 3 (RIN 8.8)


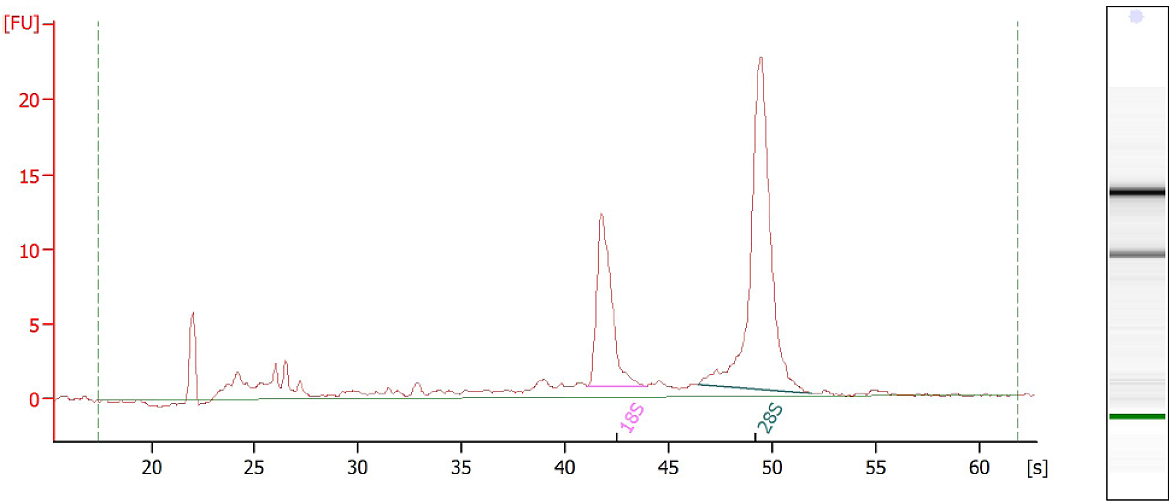


PAH Sample 4 (RIN 8.3)


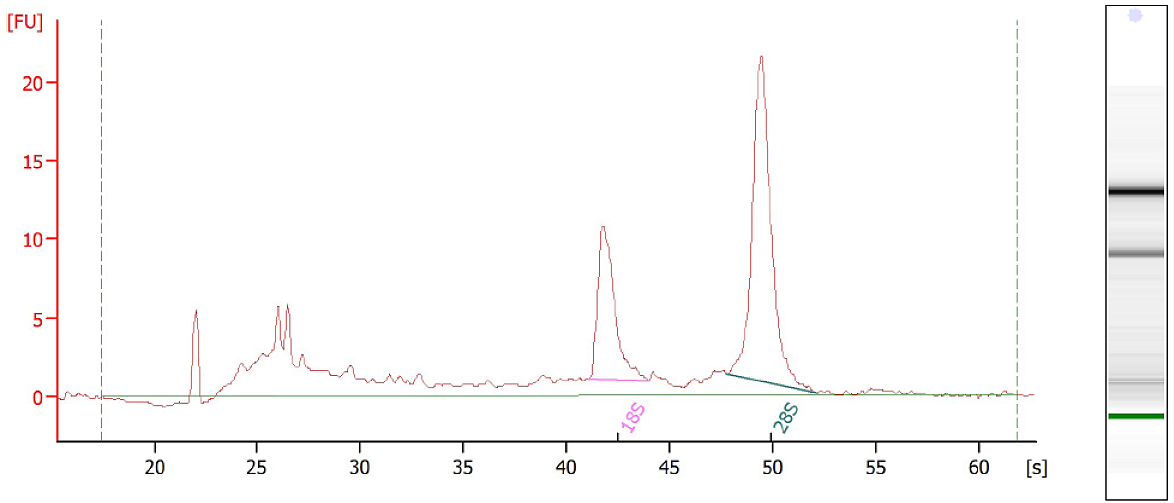


PAH Sample 5 (RIN 8.5)


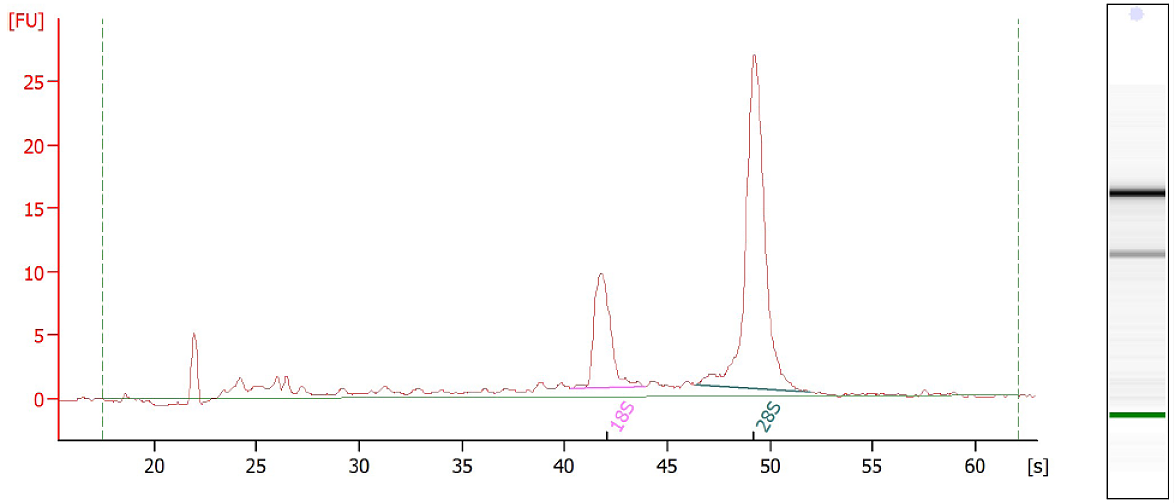


PAH Sample 6 (RIN 8.6)


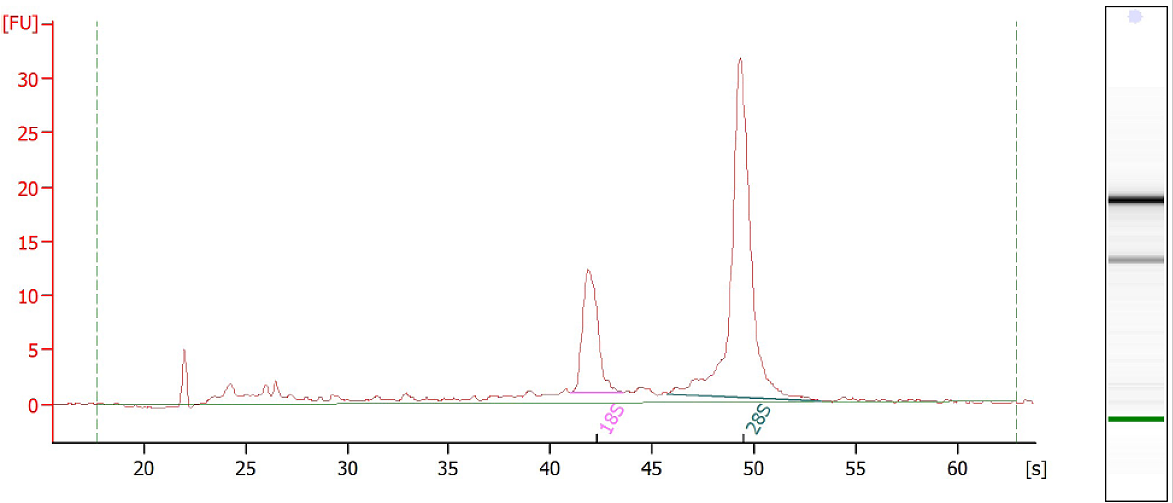

Supplement: S2 Supporting Information — (DOCX) [file pone.0211909.s002.docx]
