# Supplementary figures and images for: Isolating pulmonary microvascular endothelial cells ex vivo: Implications for pulmonary arterial hypertension, and a caution on the use of commercial biomaterials
Source: PLoS One. 2019 Feb 27;14(2):e0211909. doi: 10.1371/journal.pone.0211909 (PMC6392245; doi:10.1371/journal.pone.0211909)

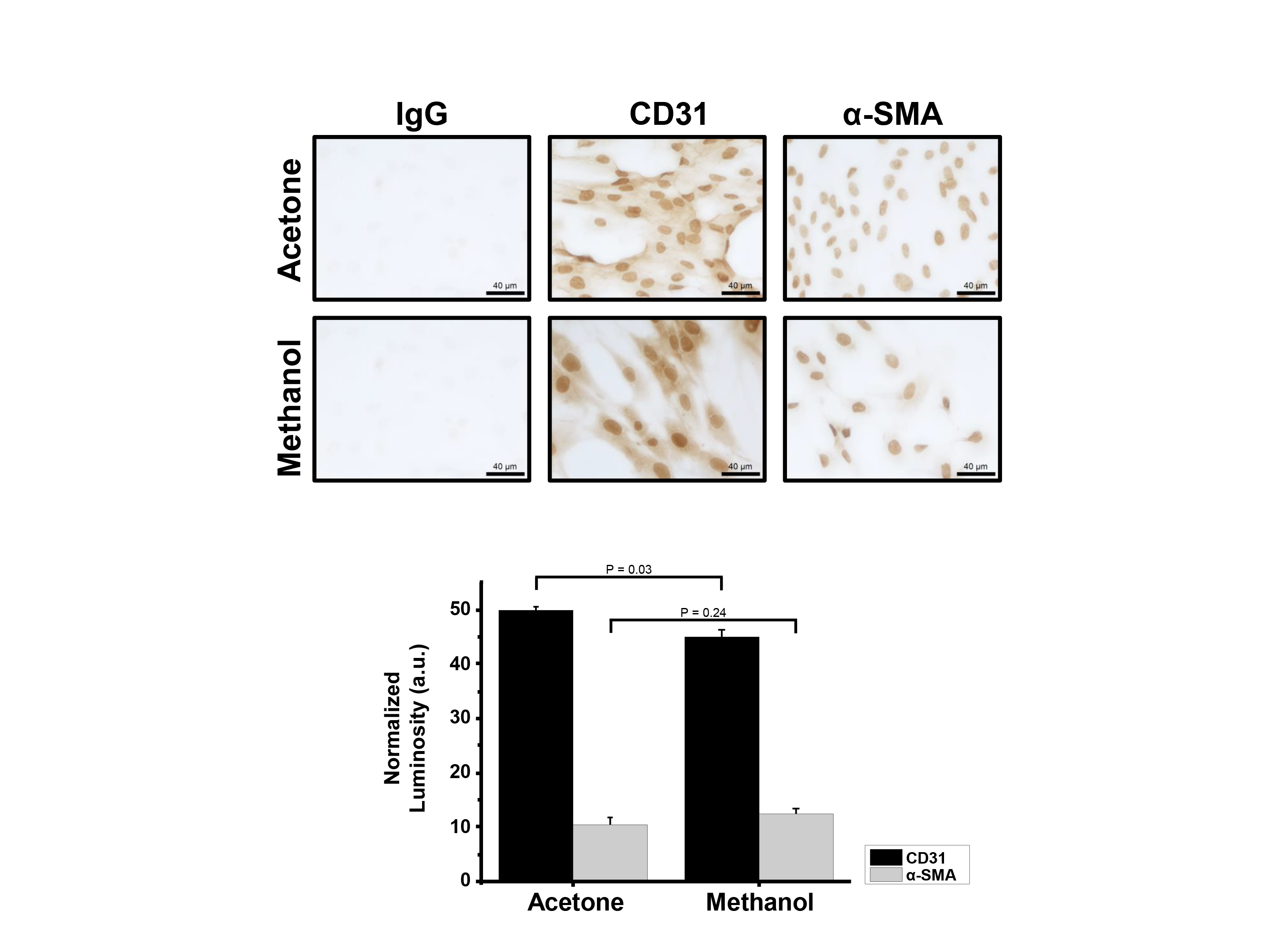

Supplement: S1 Fig — Peripheral rat lung tissue was treated with mechanical and enzymatic dissociation, and the cell pellet was cultured in endothelial-selective medium. Presumed rat PMVECs were fixed in acetone or methanol and analyzed using anti-CD31 Ab #1 and anti-α-smooth muscle actin Ab # 7 immunocytochemistry. Luminosity was normalized to IgG (Ab #3). Representative images shown. a.u., arbitrary units. Student’s unpaired t-test. Means ± SE, N = 3/condition. (TIF) [file pone.0211909.s003.tif]

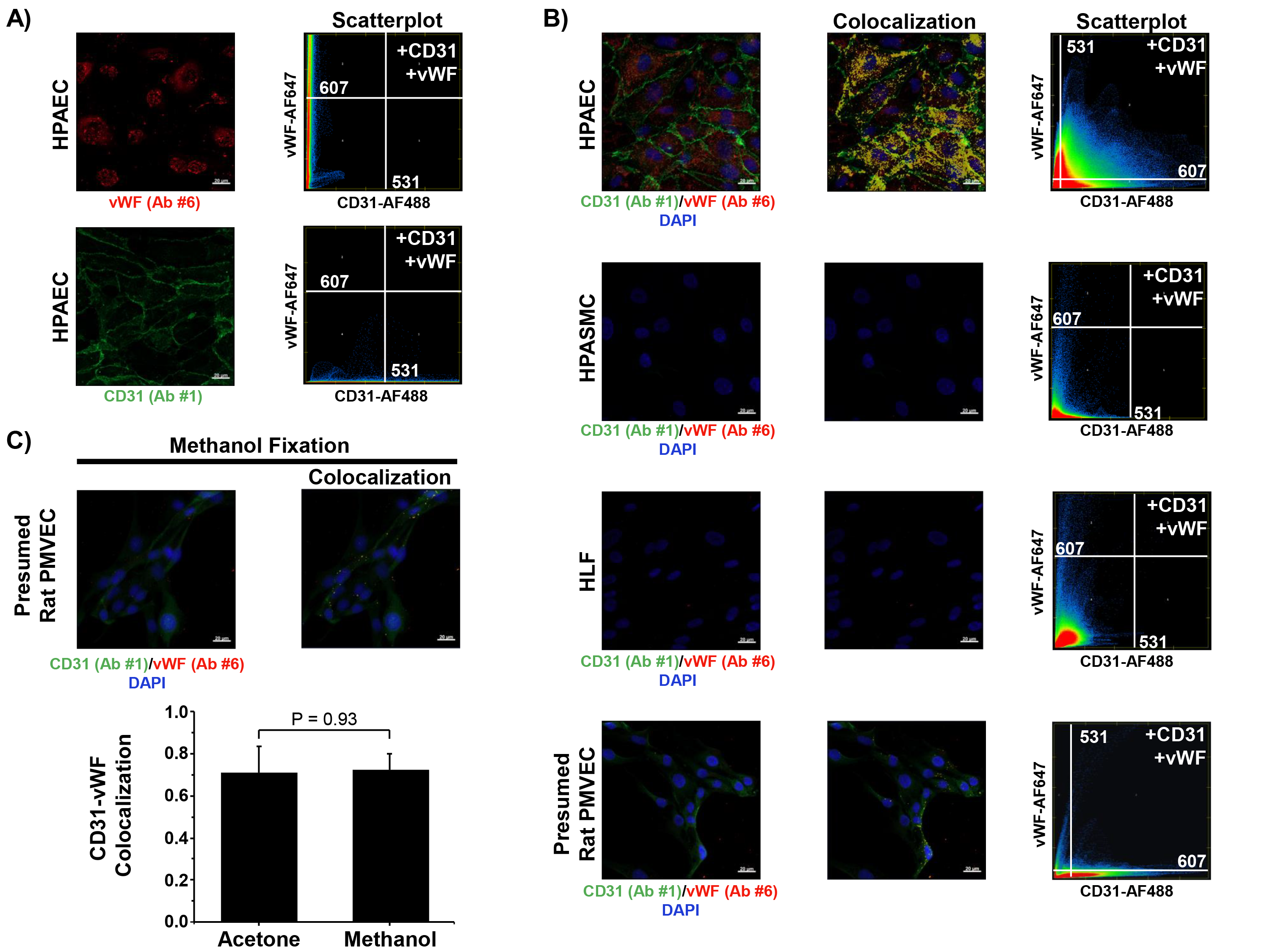

Supplement: S2 Fig — (A) Human pulmonary artery endothelial cells were labeled with either anti-CD31 Ab #1 or anti-von Willebrand Factor Ab #6 and analyzed using confocal microscopy to determine colocalization thresholds. (B) Peripheral rat lung tissue was subjected to mechanical and enzymatic dissociation, and the cell pellet was cultured in endothelial-selective medium. Presumed rat PMVECs, human pulmonary artery endothelial cells, human pulmonary artery smooth muscle cells, and human lung fibroblasts were fixed in acetone and co-labeled with anti-CD31 Ab #1 and anti-von Willebrand Factor Ab #6 and colocalization was measured using the thresholds established in panel (A). To enhance visualization, regions of colocalization are emphasized using a false-colored yellow overlay. (C) Meaningful differences in CD31-vWF colocalization were not observed between methanol and acetone fixation of presumed rat PMVECs. Representative images and scatterplots shown. AF 488, Alexa Fluor 488; AF 647, Alexa Fluor 647. Student’s unpaired t-test. Means ± SE, N = 3/condition. (TIF) [file pone.0211909.s004.tif]

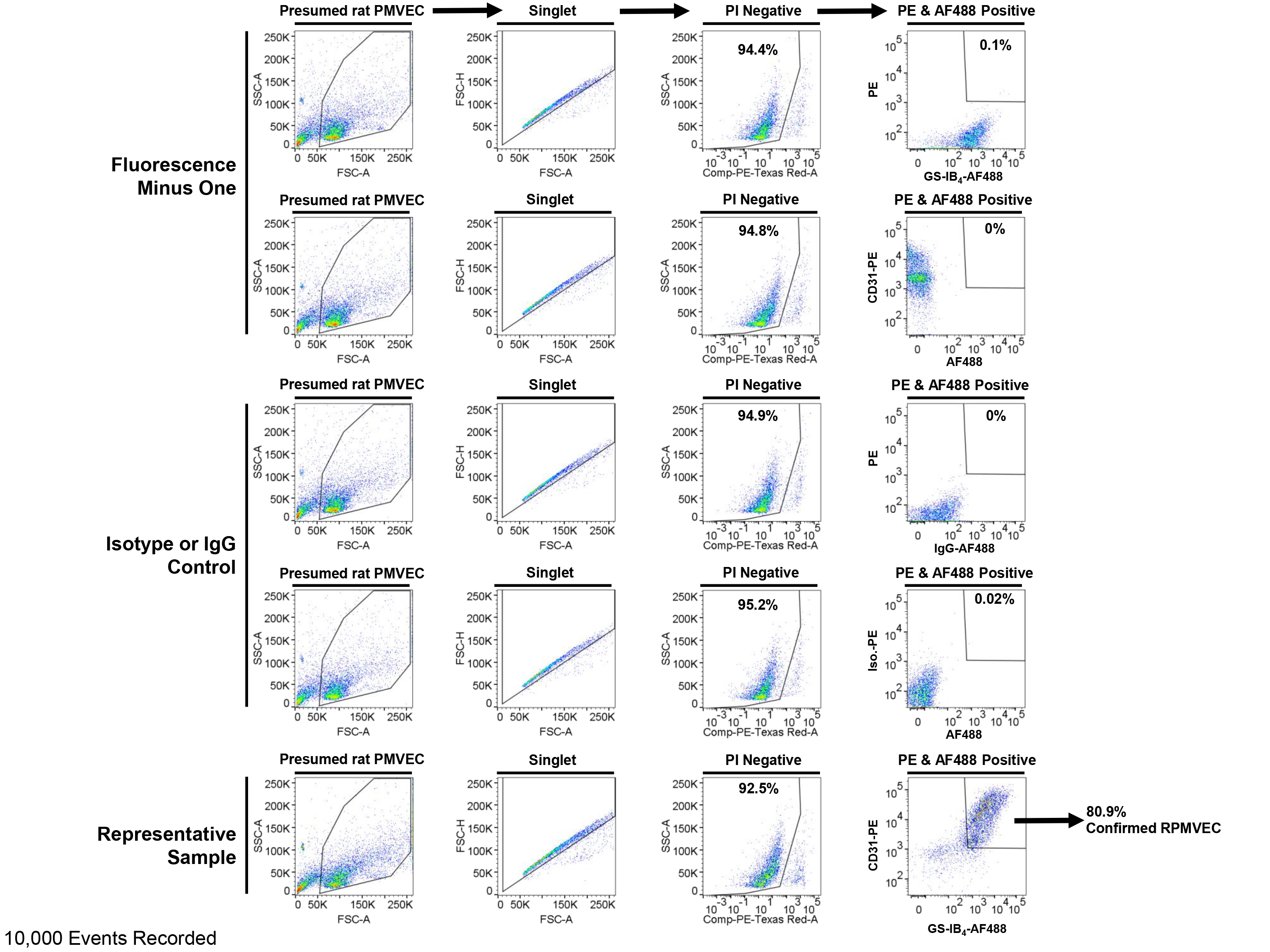

Supplement: S3 Fig — Presumed rat PMVECs were isolated without cell culture by mechanical and enzymatic digestion and immunomagnetic bead selection for CD31. Presumed rat PMVECs were labeled with anti-CD31 Ab #20 (conjugated to phycoerythrin) and Griffonia simplicifolia isolectin 1-B4 (conjugated to Alexa Fluor 488) and analyzed by flow cytometry. Fluorescence minus one controls were used to establish gates. Isotype or IgG control confirmed the specificity of cell labeling by Griffonia simplicifolia isolectin 1-B4. Viability was assessed by propidium iodide. Representative plots shown. AF 488, Alexa Fluor 488; AF 647, Alexa Fluor 647; FSC-H, forward scatter-height; PE, phycoerythrin; PI, propidium iodide; SSc-A, side scatter-area. (TIF) [file pone.0211909.s005.tif]
